# Histone Deacetylase 8 Is Required for Centrosome Cohesion and Influenza A Virus Entry

**DOI:** 10.1371/journal.ppat.1002316

**Published:** 2011-10-27

**Authors:** Yohei Yamauchi, Heithem Boukari, Indranil Banerjee, Ivo F. Sbalzarini, Peter Horvath, Ari Helenius

**Affiliations:** 1 Institute of Biochemistry, ETH Zurich, Zurich, Switzerland; 2 Institute of Theoretical Computer Science and Swiss Institute of Bioinformatics, ETH Zurich, Zurich, Switzerland; 3 Light Microscopy Center, Department of Biology, ETH Zurich, Zurich, Switzerland; Johns Hopkins University - Bloomberg School of Public Health, United States of America

## Abstract

Influenza A virus (IAV) enters host cells by endocytosis followed by acid-activated penetration from late endosomes (LEs). Using siRNA silencing, we found that histone deacetylase 8 (HDAC8), a cytoplasmic enzyme, efficiently promoted productive entry of IAV into tissue culture cells, whereas HDAC1 suppressed it. HDAC8 enhanced endocytosis, acidification, and penetration of the incoming virus. In contrast, HDAC1 inhibited acidification and penetration. The effects were connected with dramatic alterations in the organization of the microtubule system, and, as a consequence, a change in the behavior of LEs and lysosomes (LYs). Depletion of HDAC8 caused loss of centrosome-associated microtubules and loss of directed centripetal movement of LEs, dispersing LE/LYs to the cell periphery. For HDAC1, the picture was the opposite. To explain these changes, centrosome cohesion emerged as the critical factor. Depletion of HDAC8 caused centrosome splitting, which could also be induced by depleting a centriole-linker protein, rootletin. In both cases, IAV infection was inhibited. HDAC1 depletion reduced the splitting of centrosomes, and enhanced infection. The longer the distance between centrosomes, the lower the level of infection. HDAC8 depletion was also found to inhibit infection of Uukuniemi virus (a bunyavirus) suggesting common requirements among late penetrating enveloped viruses. The results established class I HDACs as powerful regulators of microtubule organization, centrosome function, endosome maturation, and infection by IAV and other late penetrating viruses.

## Author Summary

Histone deacetylases (HDACs) are generally associated with the epigenetic regulation of gene expression in the nucleus, but some have been shown to possess cytoplasmic functions. While analyzing the role of cell factors in influenza A virus entry into host cells, we observed that depletion of members of the class I HDAC family dramatically affected the efficiency of infection. Depletion of HDACs 8 and 3 decreased, and depletion of HDAC1 elevated the efficiency of entry. For HDAC1 and 8, this could be traced back to opposing effects on the architecture of centrosomes and consequences on microtubule organization. HDAC8 depletion caused the centrosomes to split and move away from each other. The microtubules were disorganized, and endosomes failed to move to the perinuclear region of the cell. Endocytosed viruses did not penetrate because the endosomes dispersed throughout the cytoplasm and did not acidify properly. In contrast, when HDAC1 was depleted, fewer centrosomes were split, and endosome transport and acidification became more efficient. Taken together, our results showed for the first time that class I HDACs play a role in the organization of the microtubule network, in endosome maturation, and in the entry of influenza and other late penetrating viruses into host cells.

## Introduction

To enter their host cells, the majority of animal viruses take advantage of the cell's endocytic machinery. After uptake, penetration of the viruses or their capsids into the cytosol generally occurs from early or late endosomes (EEs or LEs). Since endocytosis and endosome maturation are complex and tightly regulated activities, successful entry and infection relies on numerous cellular factors and processes. This is clearly illustrated by recent high-throughput siRNA screens that have identified hundreds of host cell genes required for infection by different viruses [1], [2]. The starting point for our study was a 7000 druggable-genome RNAi screen performed on the influenza A X31 strain (A/Aichi/2/68) (H3N2) in A549 cells suggesting that histone deacetylases (HDACs) are modulators of early infection.

IAVs are enveloped animal viruses with a segmented, negative-sense RNA genome. Point mutations, reassortment, and interspecies transmission cause recurrent epidemics and global pandemics in humans, birds, and animals [3]. At the cellular level, infection begins by virus binding to sialic acid residues on cell surface glycoproteins and lipids followed by internalization either via clathrin-mediated endocytosis or a clathrin-independent, macropinocytosis-like uptake process [4], [5], [6], [7]. The virus particles are transported into the endosome system. Penetration of the genome into the cytosol is mediated by the hemagglutinin (HA) glycoprotein, an acid-activated membrane fusion factor [8]. The low pH threshold for HA activation (pH 5.4-4.9) dictates that penetration by membrane fusion takes place in LEs or endolysosomes usually in the perinuclear region of the cell [9], [10]. After penetration, the matrix protein (M1) dissociates, and the viral ribonucleoproteins (vRNPs) are imported via nuclear pore complexes into the nucleus where replication and transcription take place [11], [12].

The centrosome is the major microtubule organizing center (MTOC) of animal cells. Centrosomes bind more than 100 regulatory proteins, whose identities suggest roles in a multitude of cellular functions [13]. By nucleating and anchoring microtubules (MTs), centrosomes influence most MT-dependent processes, including organelle transport, cell shape, polarity, adhesion, motility, and division. After duplication of the two centrioles during S phase [14], the two resulting centriole doublets continue to function as a single MTOC until they separate at the onset of the mitosis.

Acetylation is a reversible post-translational modification that neutralizes the positive charge of lysines, changing protein function in diverse ways [15]. It plays a central role in the epigenetic regulation of gene expression through modification of histone tails by histone acetyltransferases (HATs) and histone deacetylases (HDACs) [16]. Acetylated proteins are typically subunits of large macromolecular complexes involved in processes such as chromatin remodeling, cell cycle regulation, splicing, nuclear transport, MT stability, and actin nucleation. With more than 1700 substrate proteins identified by proteomic analysis, the regulatory scope of lysine acetylation is broad [17].

HDACs are classified into three subclasses [18]. In this study, we focused on class I HDACs (HDAC1, 2, 3, 8) and their role in infection. We found that some of them increased or decreased IAV's productive entry by modulating endocytosis, by affecting the properties of the MT network, and by influencing the maturation of endosomes. HDAC8 was found to support infection by promoting LE/LY motility and MT organization at the centrosome, and by increasing centrosome cohesion. In contrast, HDAC1 decreased centrosome cohesion and suppressed IAV infection. The two enzymes thus displayed opposing effects on IAV entry by controlling endosome function through changes in the MT network and the centrosome.

## Results

### HDAC8 is required for efficient IAV entry

In a high-throughput RNAi screen, HDAC8 emerged as one of the proteins required for efficient infection of influenza A X31 strain (A/Aichi/2/68) (H3N2) in A549 cells. The readout for this screen was the expression of newly synthesized viral nucleoprotein (NP). To validate this finding and expand it to other class I HDACs, we adopted the siRNA silencing approach and tested several oligonucleotides against HDACs 1, 2, 3 and 8 for effects on IAV infection. The specificity and efficacy of the siRNAs were confirmed at the protein level by Western blotting and at the mRNA level by real-time PCR (Figure S1A and B). The efficiency of protein depletion for HDACs 1, 3 and 8 was 90%, 80% and 95%, respectively (Figure S1A). The effects of the siRNAs were specific in that each of them only induced depletion of the intended HDAC (Figure S1B).

Seventy-two hours after transfection with these siRNAs, cells were infected with influenza A X31 strain (A/Aichi/2/68) (H3N2), and 10 h later, the fraction of infected cells was quantified by immunofluorescence staining against the newly synthesized NP. For imaging, automated microscopy was used, and the fraction of infected cells quantified with a MATLAB program using an algorithm developed in our lab [19]. To allow detection of an increase as well as a decrease in the number of infected cells, conditions were adjusted so that 20% of the cells were infected in cells transfected with a control oligonucleotide (All*Neg).

**Figure 1:**
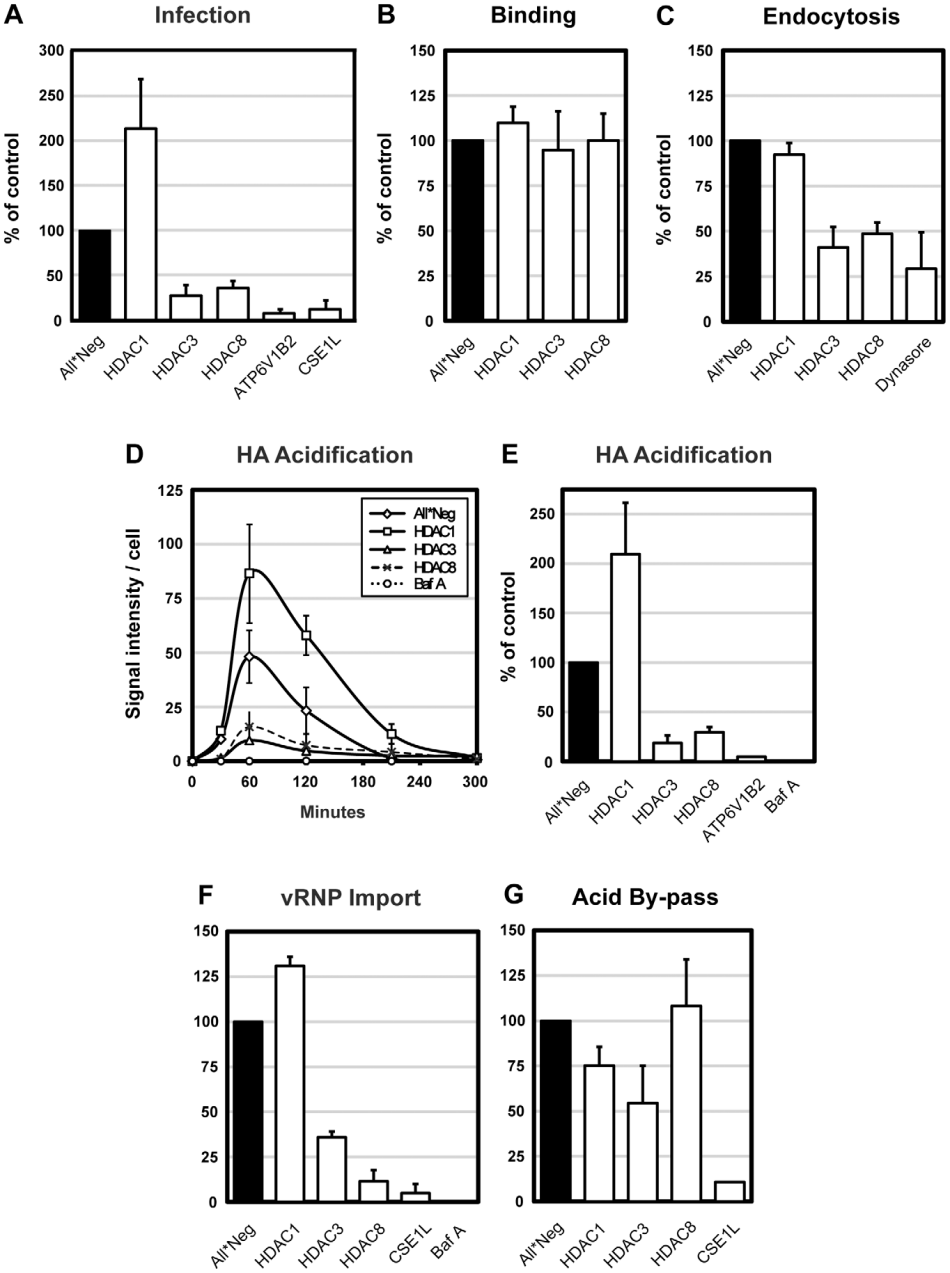
HDAC8 is required for IAV X31 infection, endocytosis and HA acidification. (A) IAV X31 infection assay. Infection was quantified in A549 cells depleted of HDAC1, 3, 8, vATPase subunit ATP6V1B2 and CAS (CSE1L) for 72 h. Cells were infected with X31 at TCID50 = 7.5×10^3^/ml in infection medium (D-MEM, 50 mM HEPES pH 6.8, 0.2% BSA). After fixation, cells were incubated in permeabilization buffer (0.1% saponin, 1% BSA in PBS) and stained for NP by indirect IFA with monoclonal antibody HB-65. Nuclei were stained with Hoechst. Images were acquired by automated microscopy and the fraction of cells expressing NP was quantified using a MATLAB-based infection scoring procedure (The MathWorks, Inc.) and normalized to control (All*Neg) cells. Twenty % of control cells are infected under these conditions. (B–F) IAV X31 entry assays. A549 cells were depleted of HDAC1, 3, 8, and where indicated, ATP6V1B2 or CAS (CSE1L). For all experiments, cells were first bound with TCID50 = 2.4×10^6^/ml virus for 1 h at 4°C. (B) Binding assay. After binding, cells were washed and fixed. Viral particles bound to the cell surface were stained by indirect IFA with anti-X31 Pinda antibody. (C) Endocytosis assay. After binding, cells were washed and warmed to 37°C, and fixed after 30 min. After blocking extracellular hemagglutinin (HA) antigens with the Pinda antibody, endocytosed virus particles were stained by indirect IFA with an HA1-specific monoclonal antibody. To block dynamin-dependent endocytosis, cells were pretreated with 120 µM dynasore 30 min prior to and during the endocytosis assay. (D) HA acidification assay. After binding, cells were washed and, or warmed to 37°C and fixed after 30, 60, 120, 210 and 300 min. Acidified HA was stained by indirect IFA with anti-A1, a monoclonal antibody that recognizes the acid-induced conformation of HA. Bafilomycin A was added to control cells to block endosome acidification. (E) Cumulative HA acidification up to 5 h was calculated and normalized to control cells. (F) vRNP nuclear import assay. After binding, cells were washed, warmed to 37°C in the presence of 1 mM cycloheximide and fixed at 5 h. Incoming NP (vRNP) was stained by indirect IFA with HB-65. Bafilomycin A was added to control cells to block endosome acidification. (G) Acid-mediated by-pass of endocytosis. Cells were bound with TCID50 = 1×10^6^/ml virus for 1 h at 4°C, washed, followed by incubation for 2.5 min at 37°C in low pH medium (pH 5.0) to induce fusion of the viral envelope and the plasma membrane, thereby releasing vRNPs directly into the cytosol. Cells were incubated for another 12 h in medium buffered to pH 7.4 containing 20 mM NH_4_Cl to block acidification of endosomes. Infection was quantified as in the infection assay. All data are represented as mean ± SEM.

Depletion of HDAC3 and HDAC8 was found to reduce IAV infectivity to 28% and 36% of control, respectively, whereas depletion of HDAC1 increased infectivity more than two-fold (Figure 1A). Depletion of HDAC2 had no effect (not shown). In cells depleted of a subunit of the vATPase (ATP6V1B2) responsible for acidification of endosomes, and CAS (CSE1L), a factor required for nuclear import of influenza vRNPs, infection was almost completely inhibited. In HeLa ATCC cells, HDAC1, 3, and 8 silencing had similar effects on infection as in A549 cells (Figure S1D).

To determine which steps in the infection cycle were affected, we used a series of quantitative, microscopy-based assays that allowed us to follow the progress of incoming virus particles by monitoring binding to the plasma membrane, internalization by endocytosis, conversion of the HA to its acid-induced conformation, and import of vRNPs into the nucleus. The results in figure 1B–G showed that the increase in infection caused by HDAC1 depletion correlated with a doubling in the efficiency of virus exposure to low pH judging by the increased conversion of HA to the acid-induced conformation detected using a specific monoclonal anti-HA antibody (Figure 1D, E). The import of vRNPs to the nucleus was correspondingly increased about 1.3-fold (Figure 1F). Since virus binding and internalization were unaffected (Figure 1B, C), this indicated that the fraction of endocytosed viruses undergoing acidification and productive penetration was elevated.

In cells depleted of HDAC8 or HDAC3, the loss of infectivity was explained by a dual effect on endocytosis and acidification. When measured 30 min after uptake, endocytosis of IAV was reduced to 48 and 41% (Figure 1C). Total acidification was reduced to 30% and 17%, compared with controls, respectively, meaning that roughly half of endocytosed viruses underwent acid exposure (Figure 1E). Consequently, the nuclear import of vRNPs was also reduced (Figure 1F). When dynasore, an inhibitor of dynamin [20] was used, the level of internalization dropped to 29% (Figure 1C). Bafilomycin A (a vATPase inhibitor) blocked acid conversion and nuclear import almost completely (Figure 1E, F). The kinetics of the conversion of HA to the acid-induced conformation was not affected by depletion of any of the HDACs. The acid HA peaked around 1 hour after warming, followed by a decline caused most likely by degradation of the HA in endolysosomes. In HDAC8- and HDAC3-depleted cells, the amount of acidic HA remained low at all time points (Figure 1D).

The assays indicated that the drop in influenza infectivity after HDAC depletion involved pre-penetration steps in the endocytic pathway. This was confirmed for HDAC1 and HDAC8 by inducing infection without the need for the virus to undergo endocytosis. Such by-pass was achieved by adding low pH medium to cells with bound virus, thus inducing fusion of the viral envelope directly with the plasma membrane [21]. In HDAC1-depleted cells, no increase in infectivity was observed compared to control cells, and no loss of infectivity was seen in HDAC8-depleted cells (Figure 1G). Cells depleted of CAS served as a post-penetration control: here the block could not be by-passed. We concluded that the effects of HDAC8 and HDAC1 involved pre-penetration steps in the endocytic pathway. However, the by-pass in HDAC3-depleted cells only partially restored infectivity (55% of control), implying that this enzyme was involved in both pre- and post-penetration steps (Figure 1G). We did not study HDAC3 further.

Taken together, the results indicated that class I HDACs participate in the regulation of the endocytic pathway, especially the pathway from EEs to LYs. Perturbation of HDACs had major consequences on the efficiency of IAV entry. Productive entry was enhanced after HDAC1 depletion and inhibited after HDAC8 or HDAC3 depletion. In HDAC8- and HDAC3-depleted cells, the endocytic internalization and acid-conversion of the virus were inefficient with the outcome that few vRNPs reached the nucleus. In HDAC1-depleted cells, primary endocytosis was normal but acid exposure, penetration, and vRNP delivery to the nucleus were dramatically enhanced.

### HDAC8 depletion causes dispersal of Golgi and LE/LYs

**Figure 2:**
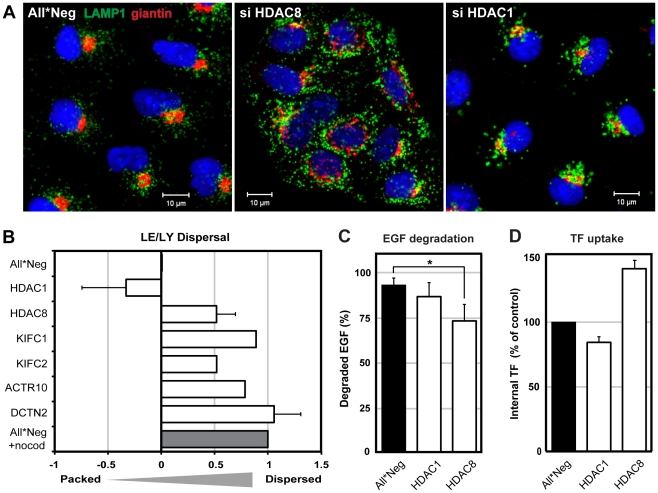
HDAC8 depletion induces dispersal of LE/Lys. (A) Localization of Golgi complex (giantin, red) and LE/LYs (LAMP1, green) in control (All*Neg), HDAC8-depleted (si HDAC8) and HDAC1-depleted (si HDAC1) A549 cells. Cells were fixed and stained by indirect IFA with anti-giantin and anti-LAMP1 antibodies. Nuclei were stained with DRAQ5. (B) Determination of LE/LY dispersal index. A549 cells were depleted of HDAC1, 8, KIFC1, KIFC2, and dynactin subunits ACTR10 and DCTN2. Cells were fixed and stained by indirect IFA with anti-LAMP1 antibody. Actin was stained by phalloidin, and DNA with Hoechst. Images were acquired automatically using a 20× objective and analyzed to derive a dispersal index of LAMP1-positive endosomes. Dispersal indices were set to zero for control (All*Neg) cells, and to 1.0 for cells treated with 30 µM nocodazole for 1 h (All*Neg+nocod). Data are represented as mean ± SEM, except for KIFC1, KIFC2, ACTR10, for which the mean from 4 different siRNAs are shown (2 independent experiments). (C) EGF degradation. A549 cells were depleted of HDAC1, 8 in 96-well Matrix plates, and 48 h after depletion, starved in serum-free medium for 24 h. EGF-AF647 (200 ng/ml) was bound for 30 min on ice. After washing, the cells were warmed to 37°C in serum-containing medium to allow internalization of EGF. At 15 min and 4 h post warming, cells were washed in acid buffer (0.1 M Glycine, pH 3) for 2 min on ice to remove extracellular EGF, washed and fixed, and stained with Hoechst. EGF and nuclei were imaged automatically using a 10× objective. EGF signal intensity was quantified using ImageJ and the percentage of EGF degradation at 4 h (compared to 15 min) per nucleus is shown. Statistical significance was assessed by Student's t-test (*) p<0.05. Data are represented as mean ± SEM. (D) Transferrin (TF) uptake. A549 cells were depleted of HDAC1, 8 in 12-well plates. On the third day of depletion, cells were starved in serum-free medium for 4 h, after which TF-AF488 (5 µg/ml) was bound for 30 min on ice. After washing, cells were warmed for 10 min at 37°C, and washed in acid buffer for 2 min on ice to remove extracellular TF. TF signal per cell was analyzed by FACS and normalized to control (All*Neg) cells. Data are represented as mean ± SEM.

When we examined the distribution of organelles positive for the LE/LY marker LAMP1 in HDAC8-depleted cells by indirect immunofluorescence microscopy, we observed that instead of clustering mainly in the juxtanuclear region, the vacuoles were distributed throughout the cytoplasm (Figure 2A). The Golgi complex, visualized using giantin as a marker, was also dispersed. In contrast, HDAC1 depletion induced increased clustering of these organelles in the perinuclear region. Some of the vacuoles were larger than in controls. EEs identified by the presence of EEA1 [22] were larger in HDAC1-depleted cells than in controls but their intracellular distribution was normal (not shown).

For quantitation of the dispersion effect, we derived a dispersion index using an algorithm described in materials and methods. With a dispersion index of 1.0 for LAMP1-positive vacuoles in nocodazole-treated cells and zero for unperturbed cells, HDAC8-depleted cells had a positive index of 0.52 (Figure 2B). The depletion of two dynactin subunits (ACTR10 and DCTN2, required for MT- and dynein-dependent retrograde transport of vesicles) [23] resulted in a dispersed phenotype similar to nocodazole. Dispersed vesicles were also observed after depletion of KIFC1 (0.89) and KIFC2 (0.52), which are both minus end-directed kinesin motors [24]. Finally, cells depleted of HDAC1 had a negative index (−0.33) consistent with the increased perinuclear clustering of the vacuoles.

To determine whether the effects were general and affected endogenous cargo, we examined the fate of epidermal growth factor (EGF), which is normally transported to LYs and degraded, and transferrin (TF), which is recycled from EEs to the plasma membrane [25], [26], [27]. We observed that HDAC1 depletion had no effect on either ligand. HDAC8-depleted cells exhibited a partial decrease in the degradation of EGF (p = 0.049, Student's t-test), and intracellular TF increased by 40% suggesting an increase in endocytosis or a decrease in recycling (Figure 2C, D).

**Figure 3:**
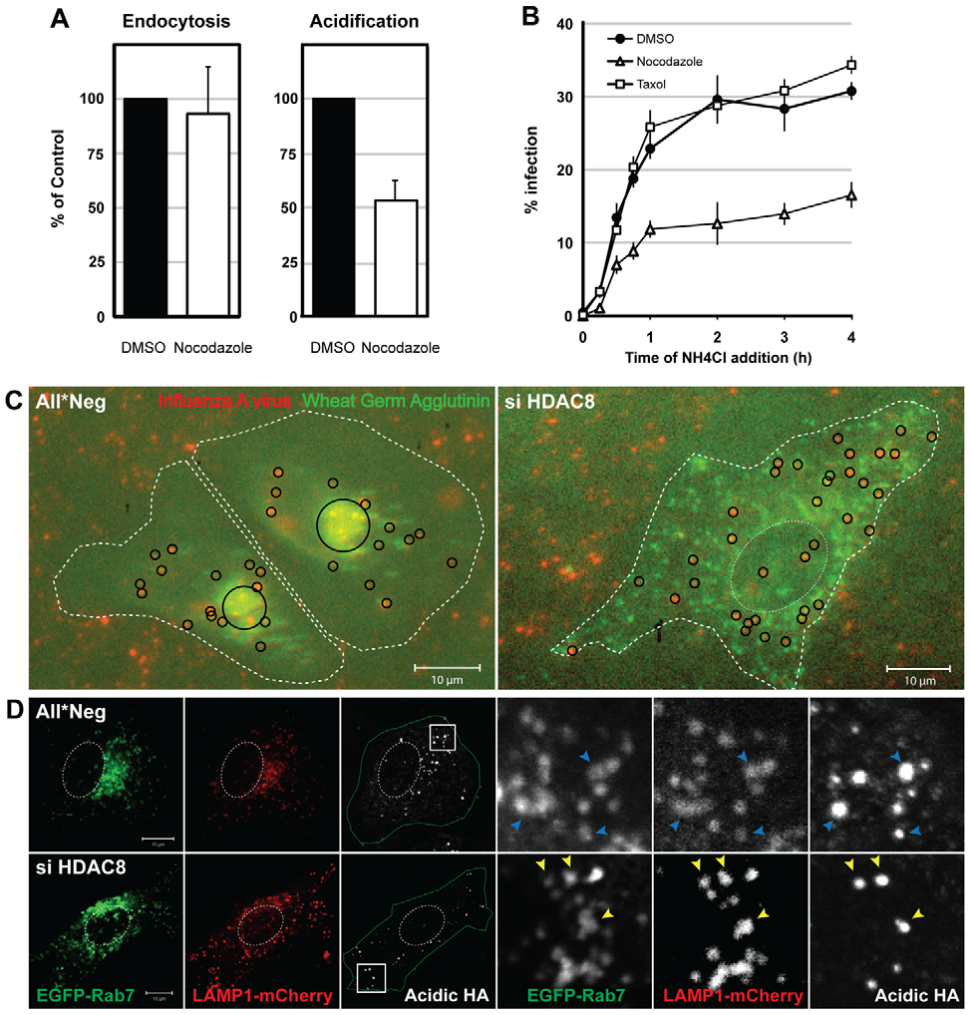
Intact MTs are required for efficient IAV X31 infection. (A) Effect of nocodazole on IAV X31 entry. A549 cells were pretreated with 30 µM nocodazole or dmso for 30 min. Virus endocytosis assay (30 min post uptake) and HA acidification assay (1 h) was performed in the presence of drug. Data are represented as mean ± SEM. (B) Washout assay of MT perturbants. A549 cells were pretreated with 30 µM nocodazole, 50 nM taxol or dmso for 30 min. Cells were bound with virus for 30 min at 4°C, washed, and warmed in the presence of the drug for 15, 30, 45 min and 1, 2, 3, 4 h, after which the medium was replaced with medium buffered to pH 7.4 containing 20 mM NH_4_Cl to block endosome acidification. Infection was analyzed at 12 h. Data are shown as mean ± SEM. (C) Live imaging of IAV X31 particles. WGA-AF647 (5 µg/ml) (shown in green pseudocolor) and R18-labeled virus (red) were bound to control (All*Neg), HDAC8-depleted (si HDAC8) A549 cells for 30 min at 4°C. After washing, cells were warmed and imaged 3 h later with a 20× objective (see also Video S1). Individual and clustered X31 particles are shown as black circles. Cell border and nucleus are indicated by dotted white lines. (D) HA acidification occurs in Rab7/LAMP1-positive LEs. Control (All*Neg), HDAC8-depleted (si HDAC8) A549 cells were transfected at 48 h after depletion with plasmids expressing Rab7-EGFP and LAMP1-mCherry. HA acidification assay was performed 24 h after the plasmid transfection. Insets are magnified and shown on the right. Blue (All*Neg) and yellow arrowheads (si HDAC8) indicate LEs positive for acidified HA.

The dispersal phenotype of LE/LYs in HDAC8-depleted cells suggested that the HDACs affected the MT system. It was therefore of interest to determine whether MT disruption and stabilization would affect infection. Nocodazole had no effect on IAV endocytosis, but reduced HA acidification by 46% and infection by roughly 50% (Figure 3A, B). Taxol had no effect. Thus by dispersing endosomes, nocodazole had an effect on infection and acidification similar to HDAC8 depletion: it reduced the infectivity of endocytosed IAV to half.

A change in endosome behavior could also be visualized by video microscopy in live cells when Alexa Fluor 647 (AF647)-labeled wheat germ agglutinin (WGA), a lectin that binds to cell surface sialic acids and glycoproteins, was allowed to be endocytosed and routed to LEs [28]. The videos showed clearly that instead of accumulating in the perinuclear region as in control cells, WGA-positive vesicles in HDAC8-depleted cells continued to move throughout the cytoplasm (Video S1). When R18-labeled IAV particles were co-internalized with WGA-AF647, they colocalized with WGA in endosomes soon after uptake. While in control cells the virus- and WGA-containing vacuoles moved into the perinuclear region, they remained peripherally distributed in HDAC8-depleted cells (Figure 3C).

The endosomal compartment in which IAV was acidified could be visualized by indirect immunofluorescence microscopy using the antibody against acidified HA. In cells transiently overexpressing EGFP-Rab7 (a marker for LEs) and LAMP1-mCherry (a marker for LE/LYs), the acidified HA signal was mainly detected in Rab7/LAMP1-positive vesicles, residing in the perinuclear region of the cell. In HDAC8-depleted cells, however, the acidified HA when present was in peripheral, Rab7/LAMP1-positive organelles corresponding most likely to LEs (Figure 3D).

These results indicated that the distribution of LE/LYs was regulated by the HDACs. HDAC8 promoted centripetal movement and perinuclear localization of LE/LYs, HDAC1 opposed the accumulation of LE/LYs in the perinuclear region. It was, moreover, possible that efficient acidification of the vacuoles was somehow linked to their location within the cytoplasm.

### HDAC8 is required for LE/LY motility

**Figure 4:**
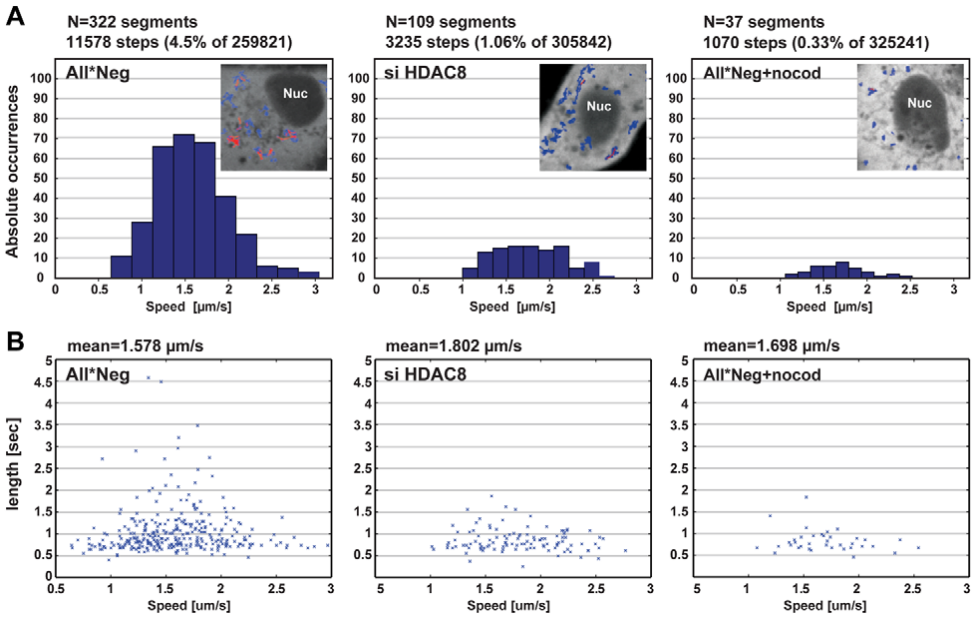
HDAC8 is required for LE/LY motility. Particle tracking analysis of endocytosed EGF. (A) Occurrence of MT-dependent directed motion (DM) and their velocity (µm/s), and (B) duration (sec) in control (All*Neg), HDAC8-depleted (si HDAC8) A549 cells and cells treated with 30 µM nocodazole for 45 min (All*Neg+nocod). (A) Videos were acquired with a Visitech Spinning Disk Confocal microscope using a 100× Objective (2000 frames, Δt = 30.53 msec), within a window of 15–30 min following uptake of EGF-AF594 (1 ng/ml). Cells were transfected with a plasmid encoding NES-2×EGFP 20 h before imaging, in order to identify the cytoplasm. EGF trajectories were extracted and classified into MT-dependent DM (red) and other types of motility (blue), as shown in the insets of panel A. DM was detected in 322 segments (comprising of 11578 steps: 4.5% of total of 259821 steps), 109 segments (3235 steps: 1.06% of 305842), 37 segments (1070 steps: 0.33% of 325241) in All*Neg (n = 6 videos), HDAC8-depleted (n = 8 videos), and nocodazole treated (n = 8 videos) samples, respectively. A total of 30–50 cells were analyzed for each condition.

To analyze the dynamics of endosome movement in more detail, we used the particle-tracking ImageJ plugin developed by Sbalzarini and coworkers and their trajectory segmentation toolbox [29], [30]. The movement of EGF-AF488-containing vesicles was recorded 15 to 30 min after internalization over 2000 frames (Figure 4A, B). Trajectories were segmented and classified according to their pattern of movement. The frequency of MT-dependent directed motion was 4.5% in control cells and 1.06% in HDAC8-depleted cells. Nocodazole treated cells had only 0.33% directed motion. The velocity of directed motion when it occurred was similar in all three cases suggesting that motor proteins were functional (Figure 4A). Of directed movements in control cells, 10.6% continued for more than 1.5 sec, whereas in HDAC8-depleted cells the value was 3.67% (Figure 4B). This meant that either the motors fell off prematurely in cells lacking HDAC8, or that the MTs were shorter or lacked stability.

### HDAC8 is required for centrosome cohesion and MT organization

**Figure 5:**
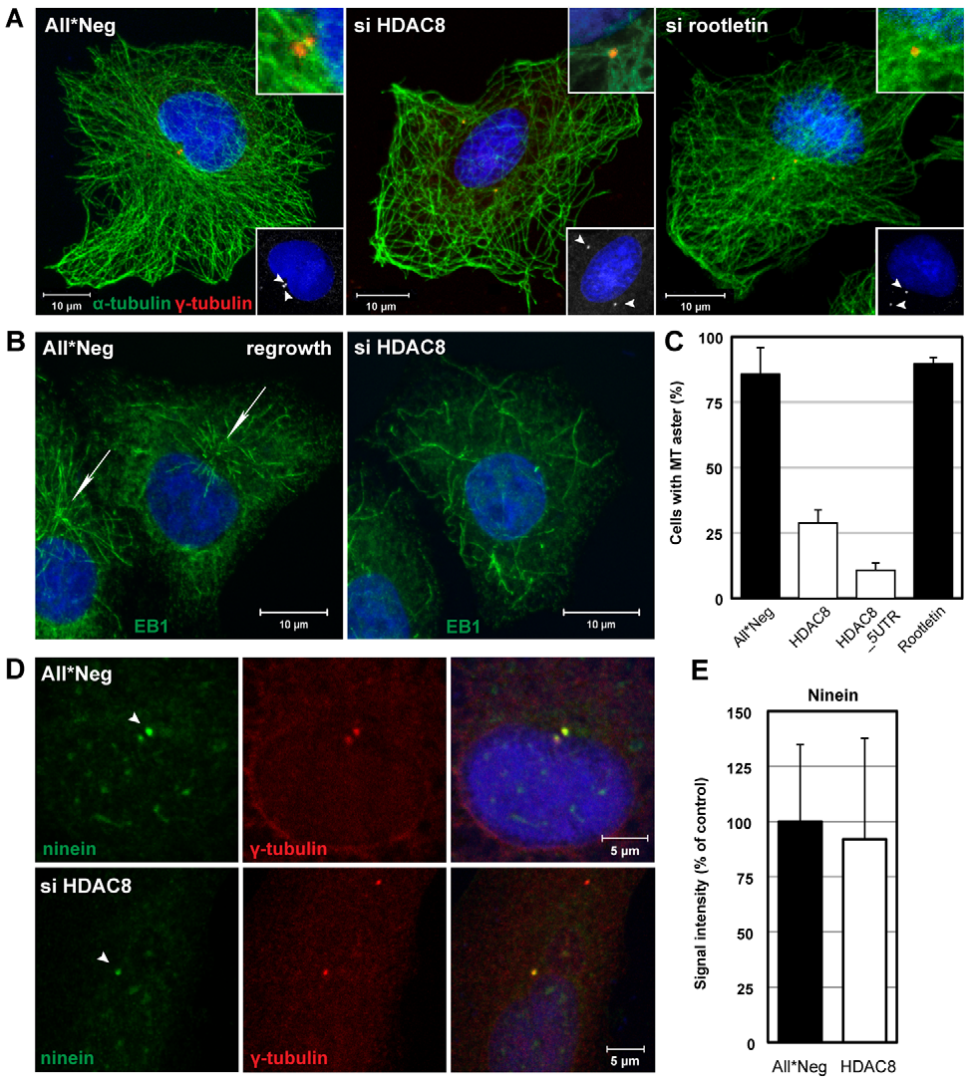
HDAC8 is required for centrosome cohesion and MT nucleation/anchorage at the centrosome. (A) Steady-state localization of α-tubulin (green) and γ-tubulin (red) in control (All*Neg), HDAC8- (si HDAC8), and rootletin-depleted (si rootletin) A549 cells. Cells were fixed in cold methanol and stained by indirect IFA. Nuclei were stained with DRAQ5. Insets at the bottom: arrowheads indicate centrosomes. Insets at the top: magnification of MT anchorage at centrosomes. (B)(C) MT regrowth assay. Control (All*Neg) and HDAC8- (si HDAC8), and rootletin-depleted (si rootletin) A549 cells were incubated on ice for 30 min to depolymerize MTs, warmed for 90 sec at 37°C to induce repolymerization and fixed immediately in cold methanol. HDAC8 was depleted using two individual oligos targeting the coding sequence (HDAC8) and the 5′UTR (HDAC8_5UTR). (B) The MT plus-end binding protein 1 (EB1) was stained by indirect IFA with the anti-EB1 antibody, and nuclei with DRAQ5. Arrows indicate MT asters. (C) Quantification of cells exhibiting MT asters. Data are represented as mean ± SEM. (D) MT anchoring protein ninein remains at centrosomes following HDAC8 depletion. Control (All*Neg) and HDAC8-depleted (si HDAC8) A549 cells were fixed in cold methanol and stained by indirect IFA with antibodies against centrosomal markers ninein and γ-tubulin. Ninein localizes with higher affinity to the mother centriole, which is indicated by an arrowhead. (E) Intensity of ninein signal at centrosomes in control and HDAC8-depleted cells. Confocal images were acquired and analyzed by ImageJ.

Immunofluorescence staining with anti-tubulin antibodies showed that, unlike nocodazole treatment, HDAC8 depletion did not eliminate the MTs, but caused disorganization of the MT system. Instead of radiating from the MTOC, the MTs were randomly oriented, criss-crossing each other in the cytoplasm (Figure 5A). In many cells, the MT network was denser at the cell periphery than in the cell center (Figure S2A).

**Figure 6:**
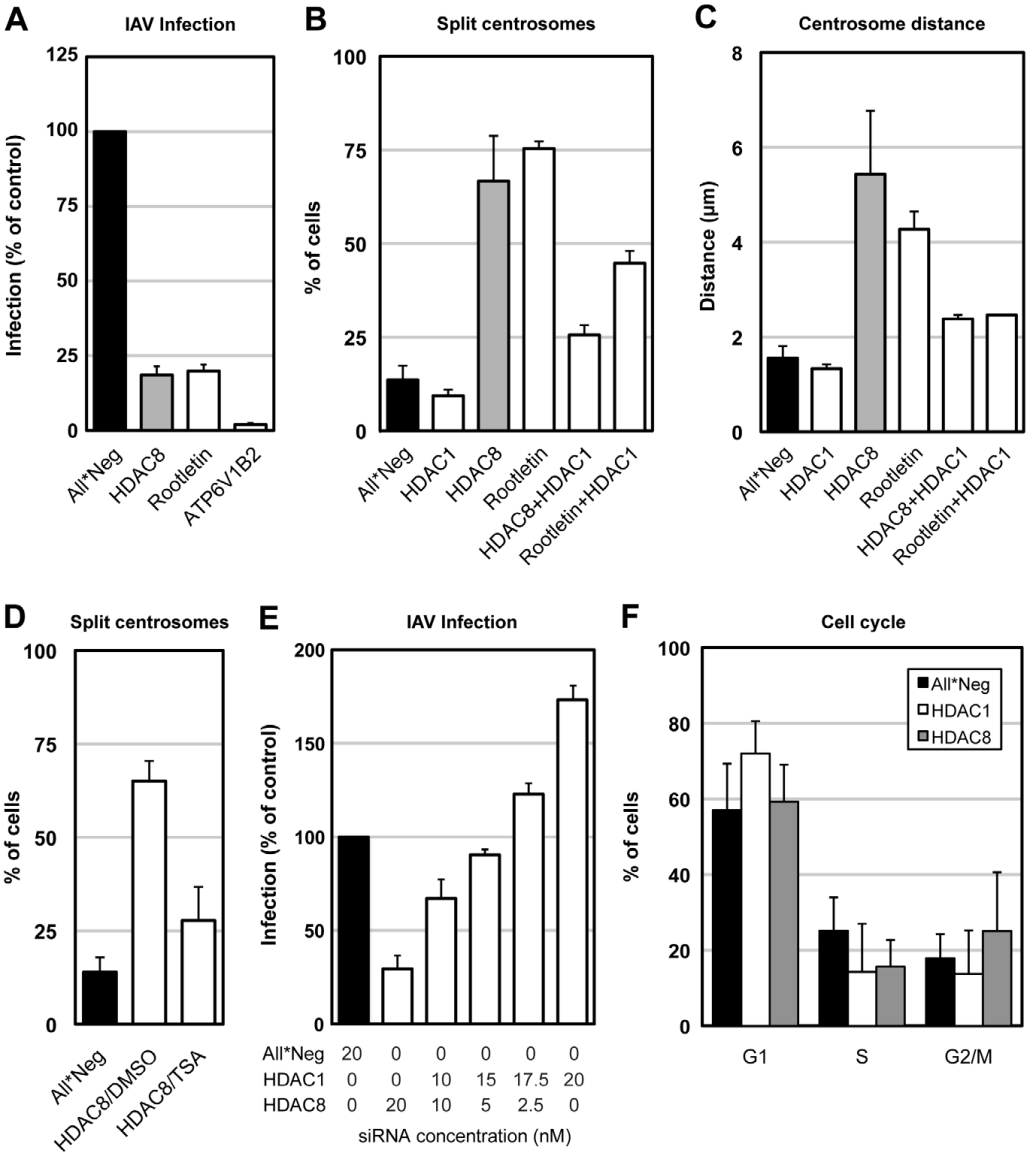
Centrosome splitting blocks IAV X31 infection and is counteracted by depletion of HDAC1. (A) Centrosome splitting per se blocks IAV X31 infection. A549 cells were depleted of HDAC8, rootletin, ATP6V1B2 and infected for 10 h. Data are represented as mean ± SEM. (B) Quantification of centrosome splitting. A549 cells were depleted of HDAC1, 8, rootletin, HDAC8/1, and rootletin/HDAC1, fixed and stained by indirect IFA with anti-γ-tubulin antibody. Maximal projections from confocal z-stack images were analyzed by ImageJ, and centrosomes that were greater than 2 µm apart were counted as split. Data are represented as mean ± SEM. (C) Quantification of centrosome distance. A549 cells were depleted of HDAC1, 8, rootletin, HDAC8/1, and rootletin/HDAC1. Centrosome distance was measured using ImageJ as in panel B. Data are represented as mean ± SEM. (D) Trychostatin A (TSA) counteracts centrosome splitting induced by HDAC8 depletion. A549 cells depleted of HDAC8 for 60 h were incubated with dmso or 5 µM TSA for 12 h, fixed and analyzed for centrosome splitting as above. Control cells were incubated with dmso for 12 h. Data are represented as mean ± SEM. (E) HDAC1 counteracts HDAC8. A549 cells were depleted of HDAC1, HDAC8 with different ratio of siRNAs (HDAC1∶HDAC8 = 0∶20, 10∶10, 15∶5, 17.5∶2.5, 20∶0; total 20 nM), and infected for 10 h. Data are represented as mean ± SEM. (F) Cell cycle analysis. A549 cells were depleted of HDAC1, 8, and trypsinized, fixed in cold EtOH, stained with 2.5 µm DRAQ5, and analyzed by FACS. The percentage of cells in G1, S or G2/M phase was quantified by FlowJo. Data are represented as mean ± SEM.

Moreover, the MTOCs were dramatically altered. Staining for centrosomes with γ-tubulin antibodies showed that instead of exhibiting two closely paired spots in the MTOC, they occurred in similar spots that were far apart, and occasionally on opposite sides of the nucleus (Figure 5A). Centrosome splitting was observed in 66.6% of HDAC8-depleted cells compared to 13.6% in the control cells (n = 500) (Figure 6B). The average distance between the two centrosomes was 5.43±1.30 µm in HDAC8-depleted cells, compared to 1.56±0.25 µm in control cells (n = 250) (Figure 6C). There was also a defect in MT anchoring to the split centrosomes; they appeared to be entirely disconnected (Figure 5A). Judging by the diffuse staining of DNA and the cell shape, these cells were in interphase, during which centrosomes do not normally split. Together with the splitting of the centrosomes, this may explain the gross redistribution and abnormal morphology of MTs.

Tubulin acetylation increases the stability of MTs [31]. To examine whether HDAC8 depletion affected the stability of tubulin, we determined the acetylation level of α-tubulin in control, HDAC1-, 6-, and 8-depleted A549 cells (Figure S3). Depletion of HDAC6, a tubulin deacetylase [32], increased tubulin acetylation by 60%, whereas HDAC8 depletion reduced it to 20% (Figure S3A, B). HDAC8 localized diffusely throughout the cell (Figure S4).

When MTs were depolymerized in the cold in HDAC8-depleted cells and allowed to regrow at 37°C, they were found to polymerize at random sites in the cytoplasm (Figure 5B). Thus, formation of MT asters after 90 sec of regrowth could be observed in only 11–29% of HDAC8-depleted cells compared to 83% of the control cells (Figure 5C). MT nucleation can be affected by the displacement of ninein, a protein normally present in both centrioles with higher affinity to the mother centriole [33]. Ninein localized to centrosomes in HDAC8-depleted cells (Figure 5D) with an efficiency of 92%, compared to control cells (Figure 5E).

### HDAC8 and rootletin are required for infection of late penetrating viruses

Was centrosome splitting the underlying reason for the inhibition of IAV infection? To test this possibility, we used siRNAs to deplete cells of rootletin, a protein present in centriole-associated fibers, the depletion of which causes centrosome splitting [34]. In rootletin-depleted cells, we observed centrosome splitting in 75.3% compared to 13.6% of control cells (n = 500) (Figure 6B). The average distance between the two centrosomes was 4.27±0.37 µm compared to 1.56±0.25 µm in control cells (n = 250) (Figure 6C). The only major difference compared with HDAC8-depleted cells was that the effects on MT organization were less dramatic under rootletin depletion (Figure 5A, S2A). LE/LYs gave an intermediately dispersed phenotype (Figure S2B). The formation of MT asters after 90 sec of regrowth was unaffected (Figure 5C). In other words, the split centrosomes were apparently able to connect with MTs.

When the effect of rootletin depletion on IAV infection was tested, we found that infection was reduced to 20% of controls (Figure 6A). Virus endocytosis was unaffected, but HA acidification was reduced to 40% of normal (not shown). Thus, by causing centrosome splitting by an independent mechanism, it was possible to induce a block in infection similar, but not identical, to that caused by HDAC8 depletion. It was apparent that by inducing the separation of centrosomes, the loss of either HDAC8 or rootletin caused changes in the MT system of the cell that resulted in incomplete endosome maturation and reduced entry of IAV.

The results observed after HDAC1 depletion seemed to support a correlation between centrosome architecture and infection. HDAC1 depletion reduced centrosome splitting to 9.3% compared to 13.6% in control cells (n = 500) (Figure 6B), and shortened the average distance between centrosomes to 1.3±0.09 µm (n = 200) (Figure 6C).

Interestingly, the centrosome splitting caused by HDAC8 depletion could be reduced to 25.6% and the inter-centrosomal distance to 2.4±0.1 µm by co-depletion of HDAC1 (n = 200) (Figure 6B, C). Depletion of both HDAC1 and HDAC8 was sufficient under both single and co-depletion conditions (Figure S5). Co-depletion of HDAC1 with rootletin also reduced splitting of centrosomes from 75.3% to 44.7%, and the average centrosome distance to a mere 2.6 µm (n = 100) (Figure 6B, C). The pan-HDAC inhibitor trychostatin A (TSA) preferentially blocks HDAC1 but does not affect HDAC8 [35]. Of HDAC8-depleted cells that were treated with 5 µM TSA for 12 h, only 28% showed centrosome splitting, compared to 65% of dmso treated cells (Figure 6D). That HDAC8 and HDAC1 had opposing effects on centrosome distance and infection was further supported by double transfection experiments where the two were silenced simultaneously. When the siRNAs were mixed, a linear conversion from inhibition to activation was observed depending on the ratio of siRNAs used (Figure 6E). Finally, compared to control cells, HDAC1 depletion increased the G1 phase population from 57% to 72%, while HDAC8 depletion increased the G2/M phase population from 18% to 25% (Figure 6F). Therefore, a specific block in the cell cycle was unlikely to be the reason for MT disorganization in HDAC8-depleted cells.

We concluded that the effects of HDAC depletion on IAV infection correlated with the architecture of centrosomes. When centrosome splitting was induced, MT organization was disturbed, with the result that endosomes failed to be transported properly into the perinuclear region. Endosome acidification was not properly activated, and virus particles failed to release their vRNPs into the cytosol. In contrast, when the average centrosome distance decreased, as it did in HDAC1-depleted cells, the efficiency of acidification and infection increased. It is of interest to note that infection did not correlate with MT anchoring to centrosomes; whereas the split centrosomes in HDAC8-depleted cells were not anchored, the centrosomes in rootletin-depleted cells remained anchored to MT (Figure 5A, S2A).

### Effects on other viruses

Since viruses use different endocytic strategies and different pathways to enter cells [36], it was of interest to test whether HDAC8 or HDAC1 depletion affected other viruses. We tested vesicular stomatitis virus (VSV, a rhabdovirus), Uukuniemi virus (UUKV, a bunyavirus), and mature virions (MVs) of vaccinia virus (VACV, a poxvirus). VSV enters cells by clathrin-mediated endocytosis and the pH threshold for fusion of the viral envelope with endosomal membranes is pH 6.2 [37], [38]. UUKV uses clathrin-independent mechanisms for infectious entry and its threshold for membrane fusion is pH 5.4 [39]. VACV MVs enter cells by macropinocytosis [40].

**Figure 7:**
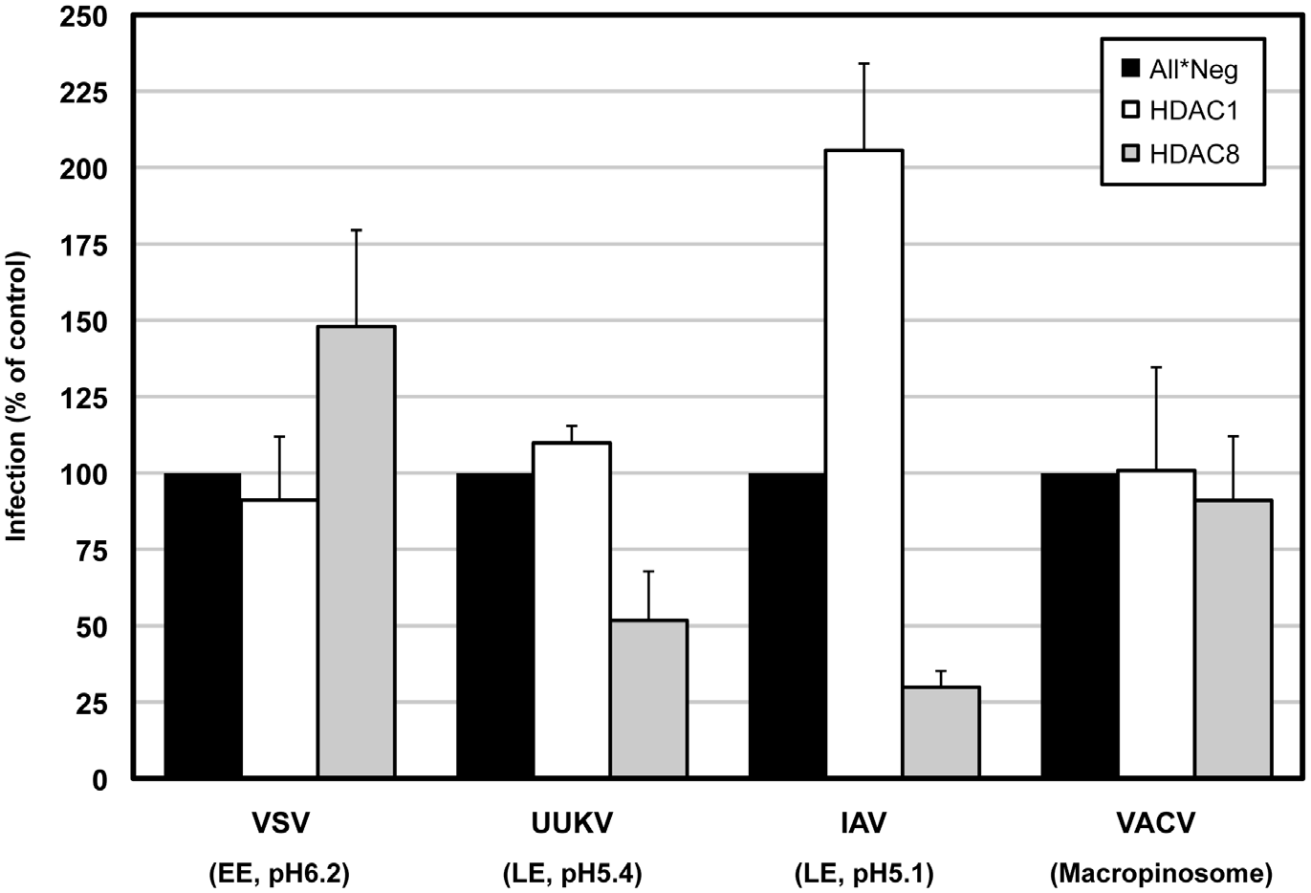
HDAC8 is required for infection of late-penetrating viruses. A549 cells were depleted of HDAC1, 8, and infected with VSV (fuses at EEs at pH 6.2), UUKV (fuses at LEs at pH 5.4), IAV (fuses at LEs at pH 5.1) and VACV MVs (fuses at macropinosomes). Infected cells were detected either by indirect IFA against a viral protein (for VSV, UUKV, IAV) or by EGFP-expression (VACV). Percentage of infected cells was quantified using a MATLAB program algorithm and normalized to control (All*Neg) cells. Virus amounts were adjusted so that 20% of cells were infected in the controls. Data are represented as mean ± SEM.

Depletion of HDAC1 had no effect on any of the viruses tested except IAV. Results were similar when cells were treated with 5 µM TSA for 4 h prior to infection (Figure S6). HDAC8 depletion reduced IAV and UUKV infection to 30% and 52%, respectively (Figure 7). VACV was unaffected. VSV infection increased by 48% confirming once again that there was no defect in clathrin-mediated uptake [37] (Figure 7). Consistent with the increase in TF accumulation, the increase in VSV infection could be due to an expansion of the EE compartment caused by the perturbation of the LE maturation program. Rootletin depletion reduced UUKV infection to 23% (Figure S7). Thus, HDAC8, rootletin and the functionality of centrosomes is critical for the infection of late penetrating viruses.

## Discussion

The most important new insights from our study were that HDAC8 and HDAC1 regulate the properties of the MT system and architecture of centrosomes in interphase cells, and that this influences the motility, distribution, and maturation of LEs and LYs. One consequence is that the infectious entry of IAV and other late penetrating viruses that require acidification to a pH of about 5.5 or lower and penetrate from LEs [41], [42], [43], are affected by the expression of class I HDACs. A role of HDAC1 or 8 in the regulation of the MT cytoskeleton in interphase cells and the maturation of endosomes has not been previously described.

Depletion of HDAC8 and HDAC3 resulted in decreased infection by IAV. HDAC8, which we analyzed more extensively of the two, is localized in the nucleus and in the cytoplasm (Figure S4). Although it is well-characterized at the molecular level, its cellular functions have remained elusive [44], [45], [46], [47]. Unlike other class I HDACs, HDAC8 does not form high-molecular weight multi-molecular complexes [15]. We found that HDAC8 depletion resulted in a two-step reduction in IAV entry. First, endocytic internalization was reduced to less than half compared to control cells. Second, of the internalized viruses only half were acidified and therefore capable of membrane fusion and penetration. Thus, the overall infection rate amounted to 15–30% of controls. That VSV infection and the internalization of TF were not inhibited, ruled out a general defect in clathrin-mediated endocytosis and the cellular translational machinery. Since IAV endocytosis occurs by two parallel pathways, a clathrin-mediated and a recently described macropinocytosis-like mechanism [7], it is possible that it is the latter pathway that is affected. The link between class I HDACs and endosome maturation may be of significance in the differentiation of cells as well as during oncogenesis, which is often associated with elevated HDAC activity. HDACs are also likely to affect the tropism and pathogenesis of influenza and other viruses that use endocytosis for entry. When HDAC inhibitors are deployed in cancer treatment, it will be of importance to consider changes in influenza virus susceptibility.

The second effect of HDAC8 depletion on IAV - the decreased exposure to pH of 5.1 or below - occurred late in the endocytic pathway. A decrease in the degradation of EGF indicated that cargo was not reaching the lysosomal compartments with normal efficiency. In HDAC8-depleted cells, internalized viruses were trapped in Rab7/LAMP1-positive vacuoles, which instead of moving along MTs to the MTOC remained dispersed in the cytoplasm. Particle tracking showed that LEs and LYs were less motile and less tightly connected to MTs, and failed to undergo sustained directional movement. The few vacuoles that contained acidified viruses had a peripheral location.

In normal cells, organized movement and acidification are part of a complex maturation program that prepares LEs for fusion with LYs. Maturation involves conversion of Rab GTPases, a switch in phosphatidylinositides, acidification, formation of intraluminal vesicles, association with dynein and dynactin, and MT-mediated transport to the perinuclear region [36], [48], [49]. These changes are coordinated and interdependent in complex ways, so that when one function is perturbed the whole program may be disrupted. Therefore it is possible that proper acidification failed to occur because the LEs did not move to the MTOC [50]. A similar situation was created when endosome movement was inhibited by disrupting MTs with nocodazole; acidification of the virus and productive infection by internalized viruses also dropped by half. The location of endosomes is determined by many factors including the presence of adaptor proteins that provide a link to the cytoskeleton, and various motors, the size and shape of the organelles, etc. [51]. Our data suggest that the properties of an endosome are affected by their location such that if they cannot move to the perinuclear region of the cell their maturation is not completed.

HDAC8 depletion had a dramatic effect on the MT system. It caused centrosome splitting, loss of tubulin acetylation and MT nucleation/anchoring. In contrast to nocodazole treatment, most MTs were still present, but since the MTOC was disrupted they were disorganized. Disruption of the MTOC may account for the loss of acetylation of tubulin. Long-term displacement of MTs from the mother centrosome may have been the reason why the centrosomes drifted apart, reminiscent of the splitting that can be induced by nocodazole treatment [52]. Aside from being split and unable to support MT anchoring, the centrosomes appeared normal after HDAC8 depletion. Important factors such as γ-tubulin and ninein were present.

The underlying reason for the reduction of IAV infection and infection with another late penetrating virus, UUKV, was most likely the loss of centrosome cohesion. The best evidence was that the induction of centrosome splitting by an independent mechanism, i.e. by the depletion of rootletin, caused dramatic reductions in infection. Rootletin depletion induced an intermediate level of LE/LY dispersal. It is possible that close centrosomes provide a more focused MT network compared to split centrosomes. It has been shown that fewer MTs radiate from centrosomes when they are apart [53].

The effects of HDAC1 depletion also supported centrosome distance as an important factor in IAV infection. HDAC1 is a class I HDAC almost exclusively localized in the nucleus, with important functions in epigenetic regulation of cell fate and cellular processes [15]. When HDAC1 was depleted, infection by IAV more than doubled. The percentage of split centrosomes decreased. Inhibition of HDAC1 function by TSA also increased IAV infection. In fact, we repeatedly observed that the shorter the distance between centrosomes in a cell population, the higher the level of infection. LEs, LYs, and the Golgi complex were tightly bundled around the MTOC, and acidification of HA was twice as efficient as in control levels.

That centrosome splitting was suppressed when HDAC8 was co-depleted with HDAC1, suggested that HDAC1 may have a role in diminishing centrosome cohesion and that HDAC8's role could be to promote MT organization by enhancing the association of MTs with the centrosome. It remains to be proven whether these HDACs work directly on centrosomes, MTs or other factors. It is possible that one or more regulators of centrosome cohesion are acetylated and serve as substrates for deacetylases. C-Nap1, a centriolar linker protein, is an interesting candidate, since it is known to be acetylated [17], and is a well known regulator of centrosome cohesion [54]. Our results show that class I HDACs are important regulators of MT organization, as well as, centrosome architecture and function, and IAV entry.

## Materials and Methods

### Cells and viruses

A549 ATCC and HeLa ATCC cells were propagated according to the ATCC guidelines. Purified influenza A X31 strain (A/Aichi/2/68) (H3N2) was purchased from Virapur (CA, USA). UUKV S23, VSV (wtVSV) (Indiana serotype) and VACV WR-GFP were used as previously described [37], [40], [55].

### Virus preparation

Purified influenza A X31 strain (A/Aichi/2/68) (H3N2) was purchased from Virapur (CA, USA). Briefly, 60 pathogen-free chicken eggs were inoculated with the virus and incubated for 2 days at 33–37°C. Harvested allantoic fluid was clarified by low speed centrifugation, and concentrated by high-speed centrifugation. The virus was further concentrated by two rounds of 10–40% sucrose gradient centrifugation. Virus bands were harvested, pooled and resuspended in formulation buffer (40% sucrose, 0.02% BSA, 20 mM HEPES pH 7.4, 100 mM NaCl, 2 mM MgCl_2_), frozen and stored at −80°C until usage (TCID50 in MDCK cells = 3.0×10^8^/ml virus).

### siRNA transfection

Cells were reverse-transfected (final 10 nM) with siRNAs (QIAGEN) in 96-well flat-bottom Matrix plates (Thermo Scientific). siRNAs were mixed with Lipofectamine RNAiMax (Invitrogen) in 30 µl OPTI-MEM (Invitrogen) for 30 min. Cells were trypsinized, counted and plated directly onto the lipofectamine mixture in 70 µl of growth medium.

### IAV X31 infection assay

A549 cells in 96-well Matrix plates were infected with TCID50 = 7500/ml virus in infection medium (D-MEM, 50 mM HEPES pH 6.8, 0.2% BSA) and fixed at 10 h in 4% formaldehyde (FA) in PBS. Cells were permeabilized in permeabilization buffer (0.1% saponin, 1% BSA in PBS) and stained for viral NP by indirect immunofluorescence with monoclonal antibody HB-65 (ATCC) (1∶10 dilution) and secondary anti-mouse antibody labeled with Alexa Fluor 488 (AF488). Nuclei were stained with Hoechst (1∶10000 dilution). Typically, 9 (3×3) or 16 (4×4) images per well were automatically acquired (see Microscopy). Cell numbers and raw infection indices for each well were determined using a MATLAB-based infection scoring procedure (The MathWorks, Inc.). With this method, cells expressing NP were counted as infected. Control cells transfected with All*Neg siRNA exhibited 15–20% infection.

### IAV X31 entry assays

Microscopy-based assays for step-wise dissection of the IAV X31 strain entry pathway were developed in our laboratory. The assays use indirect immunofluorescence for detection and are optimized for high-throughput analyses in a 96-well format (Banerjee I., Horvath P. and Helenius A., manuscript in preparation. Programs used for quantification can be downloaded at www.highcontentanalysis.org). Here we use both confocal and automated fluorescence microscopy to acquire the images and ImageJ to quantify the results.

### X31 virus binding to the cell surface

A549 cells were bound with TCID50 = 2.4×10^6^/ml virus for 1 h at 4°C, washed three times in PBS, fixed in 4% FA in PBS for 20 min, blocked in blocking buffer (1% BSA in PBS), and processed for indirect immunofluorescence with anti-X31 Pinda antibody [56] (1∶2000 dilution), phalloidin-Alexa Fluor 594 (AF594) and DRAQ5 (Biostatus Limited) to stain nuclei. Images were acquired by confocal immunofluorescence microscopy using a 40× objective and quantified using ImageJ. First, a threshold value for the Pinda signal was determined for each experiment in order to eliminate background fluorescence. The area covered by fluorescence signal above this threshold was calculated. Second, phalloidin staining was used to determine cell area. Finally, the total Pinda signal per cell area was quantified for each siRNA treatment and normalized to control (All*Neg) cells.

### X31 virus endocytosis

A549 cells were bound with TCID50 = 2.4×10^6^/ml virus for 1 h at 4°C, washed twice in infection medium (D-MEM, 50 mM HEPES pH 6.8, 0.2% BSA), and warmed to 37°C to allow virus uptake. After 30 min, cells were fixed in 4% FA for 20 min. After blocking, extracellular HA antigens were blocked overnight at 4°C with Pinda antibody (1∶500) and fixed again in 4% FA in PBS for 20 min. The cells were then permeabilized (0.1% saponin, 1% BSA in PBS) and incubated with a monoclonal antibody specific for X31 HA1 [57] (1∶100) for 2 h at room temperature. HA1 was visualized by an AF488-labeled, and Pinda by an AF594-labeled secondary antibody, actin with phalloidin-AF647. This method enables to distinguish between extracellular virus particles and internalized particles. Images were acquired by confocal microscopy and total HA1 signal per cell area was quantified and normalized as in the virus binding assay.

To block dynamin-dependent endocytosis, control (All*Neg) cells were pretreated for 30 min in either dmso or 120 µM dynasore, followed by virus binding and uptake for 30 min in the presence of drug. Cells were fixed, stained and analyzed as above.

### X31 HA acidification

A549 cells were bound with TCID50 = 2.4×10^6^/ml virus for 1 h at 4°C, washed twice in infection medium, and warmed to 37°C under the presence of 1 mM cycloheximide to block new protein synthesis. For time course experiments, cells were washed in PBS and fixed in 4% FA in PBS. For a single time point analysis, cells were washed and fixed at 1 h, where the acidification signal peaked. For detection of HA that has undergone acid-induced conformational change, cells were blocked, permeabilized and incubated with anti-A1 monoclonal antibody [58] (1∶2000) for 2 h at room temperature. Acidified HA was visualized by an AF488-labeled secondary antibody and nuclei were stained with DRAQ5. Images were acquired by confocal microscopy or automated microscopy and total signal per nucleus was quantified as above. Cumulative A1 signal over a time course of up to 5 h (t_0_ = 0, t_1_ = 30, t_2_ = 60, t_3_ = 120, t_4_ = 210, t_5_ = 300 min), was obtained by calculating the area under the curve, from the equation {(t1)(y1)+(y1+y2)(t2t1)+…+(y4+y5)(t5−t4)}×0.5.

### X31 nuclear import of vRNPs

Binding and uptake of virus was performed as in the HA acidification assay, and the cells were washed and fixed 5 h post warming. Medium containing 1 mM cycloheximide was freshly replaced at 2.5 h post-internalization. Cells were fixed in 4% FA in PBS, blocked, permeabilized and stained for viral NP with the HB-65 monoclonal antibody (1∶10) for 2 h at room temperature. NP (incoming vRNPs) was visualized by an AF488-labeled secondary antibody and nuclei were stained with DRAQ5. Images were acquired by confocal microscopy, and the percentage of cells with nuclear NP signal was counted and normalized to control (All*Neg) cells.

### Acid-mediated by-pass of X31 endocytosis

A549 cells were bound with TCID50 = 1×10^6^/ml virus for 30 min at 4°C and washed three times in infection medium. The virus was allowed to fuse in low pH medium (pH 5.0) for 2.5 min on a custom-cut metal plate warmed to 37°C in a water bath. After incubation, the cells were placed on ice, washed three times before incubation at 37°C in Stop medium (D-MEM, 50 mM HEPES pH 7.4, 20 mM NH_4_Cl) to block acidification of endosomes. The cells were washed and fixed at 12 h, permeabilized, and stained with HB-65 to detect NP. The fraction of infected cells were quantified as in the infection assay.

### Drug washout assay

Cells grown in 96-well Matrix plates were pretreated for 30 min with 30 µM nocodazole, 50 nM taxol or dmso alone, followed by binding TCID50 = 6×10^5^/ml virus for 30 min on ice. The cells were washed to remove unbound virus and warmed to 37°C in the presence of the drug. At given time points, drug-containing medium was washed out and replaced with Stop medium (D-MEM, 50 mM HEPES pH 7.4, 20 mM NH_4_Cl) to block acidification of endosomes. Cells were fixed at 12 h and analyzed as in the infection assay.

### HDAC inhibitor treatment

Cells were incubated with dmso or 5 µM trychostatin A (TSA) in normal growth medium for 4 h. The drugs were removed by washing three times in infection medium, followed by an infection assay.

### Labeling of X31 virus particles

Labeling with R18 was performed as described previously [59]. X31 virus stocks were diluted to TCID50 = 1.5×10^7^/ml in PBS and incubated in the dark with 20 µM R18 (Invitrogen) at room temperature for 1 h with continuous rocking. The mixture was filtered through a 0.2 µm filter to remove aggregated dye.

### Microscopy

Confocal fluorescence microscopy was performed with a Zeiss LSM 510 Meta system setup. Cells were either fixed for 20 min in 4% FA in PBS at room temperature for 5 min in −20°C methanol. For indirect immunofluorescence, cells were blocked in blocking buffer (1% BSA in PBS) and permeablized in permeabilization buffer (0.1% saponin, 1% BSA in PBS), followed by incubation with antibodies in permeabilization buffer. Live imaging was done with the Zeiss LSM 510 or Visitech Spinning Disk confocal microscope, using 8-well chamber-slides (Nunc). For imaging, phenol red-free D-MEM (Invitrogen) was used. Image analysis was done with ImageJ or LSM Image Browser. Automated image acquisition of 96-well Matrix plates was performed with a 10× or 20× objective using a BD Pathway 855 Bioimager (BD Biosciences) or a MD Assay Development 2 (Molecular Devices).

### Determination of LE/LY dispersal index

A549 cells seeded in 96-well Matrix plates were fixed 72 h after depletion of host cell factors and fixed in 4% FA in PBS for 20 min. After blocking and permeabilization, cells were stained for LAMP1, actin (phalloidin-AF594) and DNA (Hoechst). Sixteen images (4×4) per well were acquired automatically for all three channels using a 20× objective. First, the images were segmented to identify individual cells and to determine their phenotypic properties using the CellProfiler program [60] with custom modifications. Second, supervised machine learning was used to automatically classify cell phenotypes and a regression model was applied to automatically predict the scattering index of the phenotypes. Finally, the effects of different siRNAs or drugs were determined. The scattering index of LAMP1-positive vesicles was set to zero for control (All*Neg) cells, and to 1.0 for control cells treated with 30 µM nocodazole for 1 h. In addition to the cell types described earlier, mitotic cells and segmentation errors were distinguished using a multi-parametric non-linear analysis with the Advanced Cell Classifier program (www.cellclassifier.org). For supervised machine learning, a mixed model of combining neural networks, support vector machines, and logistic classifiers was used.

### Video and trajectory analysis

EGF-AF555 (1 ng/ml) was added to control (All*Neg) A549 cells, HDAC8-depleted (siHDAC8), or control cells treated with 30 µM nocodazole for 45 min (All*Neg+nocod). Live imaging of endocytosed EGF was performed during 15–30 min after its addition with a Visitech Spinning Disk Confocal microscope using a 100× 1.4NA Oil DIC Plan-Apochromat objective. To detect cell boundaries and the nucleus, cells were transiently transfected with NES-2×EGFP 15 h before. For a single video, 2000 frames (Δt = 30.53 msec) of one z-slice were acquired. Endocytosed EGF trajectories were extracted from the recorded videos using the ImageJ implementation of the particle-tracking algorithm [30] with *radius* = 3 pixel, *cutoff* = 0, *percentile* = 0.2, *max_displacement* = 10 pixel, and *linkrange* = 1. Only trajectories longer than 50 frames were retained for analysis. Segments of directed motion where identified in the trajectories using the MATLAB trajectory segmentation toolbox as described [29]. Speeds of directed motion were computed by dividing their total length (sum of the lengths of all trajectory segments belonging to the same directed motion) by their duration. Due to noise in the images, this rather over-estimates the true speed of motion. All post-processing was done in MATLAB R2010b (The MathWorks, Inc.).

### Quantitative Real-time PCR

SYBR green quantitative real-time PCR (qRT-PCR) was performed using the LightCycler 480 SYBR Green I Master Mix (Roche) and the RotorGeneQ thermocycler (QIAGEN). Serial dilutions of the control sample cDNA were also submitted to real-time PCR to generate a standard curve, in order to calculate the efficiency of the primers. The samples were run in triplicates, whereas –RT samples, which do not contain cDNA, due to lack of reverse transcriptase (-RT) at the cDNA synthesis step were run in duplicates. A non-template control was added to assure contamination free PCR reagents. The threshold cycle was assigned by the rotor-gene Q serial software. Quantification of the results was done with the Pfaffl method [61] and the mRNA amounts of target genes were determined relative to the C_t_-values of the control sample, and normalized to the reference gene (GAPDH).

The following primers were used; GAPDH (fwd, CTGTTGCTGTAGCCAAATTCGT; rev ACCCACTCCTCCACCTTTGA), HDAC1 (fwd, GGAAATCTATCGCCCTCACA; rev, AACAGGCCATCGAATACTGG), HDAC3 (fwd, ACGTGGGCAACTTCCACTAC; rev, GACTCTTGGTGAAGCCTTGC), HDAC8 (fwd, GGTGACGTGTCTGATGTTGG; rev, AGCTCCCAGCTGTAAGACCA).

### EGF degradation

A549 cells were depleted of HDACs in 96-well Matrix plates, and 48 h after depletion starved in serum-free medium for 24 h. EGF-AF647 (200 ng/ml) was bound for 30 min on ice. After washing, the cells were warmed in serum-containing medium at 37°C to allow internalization. At 15 min and 4 h post warming, cells were washed in acid buffer (0.1 M Glycine, pH 3) for 2 min on ice to remove non-internalized EGF, washed and fixed in 4% FA in PBS, followed by Hoechst staining. EGF and nuclei were imaged with the BD Pathway 855 Bioimager using a 10× objective. Intensity of EGF signal above background was quantified using ImageJ and the percentage of EGF degradation per nucleus at 4 h (compared to 15 min) was calculated.

### Transferrin uptake

1×10^5^ A549 cells were seeded in 12-well plates and depleted of HDAC1 or 8. On the third day of depletion, cells were starved in serum-free medium for 4 h followed by binding transferrin-AF488 (5 µg/ml) for 30 min on ice. Zero and 10 min post warming to 37°C, cells were washed in acid buffer for 2 min on ice. Transferrin signal per cell was analyzed by FACS analysis. In brief, cells were washed with PBS, detached with 200 µl trypsin per well, and collected in 1 ml PBS. The cells were centrifuged at 1,500 rpm for 5 min at 4°C to remove trypsin and re-suspended and fixed in 200 µl 4% FA in PBS for 20 min. After removal of FA by centrifugation, cells were resuspended in 300 µl FACS buffer for analysis with a BD FACSCalibur flow cytometer.

### Cell cycle analysis

A549 cells in 24-well plates were depleted of HDAC1, 8 for 72 h. The cells were washed in PBS, trypsinized and fixed in cold EtOH for 30 min at 4°C. After centrifugation of cells at 1,500 rpm for 5 min at 4°C, the supernatant was removed and the cells were stained with FACS buffer (PBS, 5 mM EDTA, 2% FCS, 0.02% NaN_3_) containing 2.5 µM DRAQ5 for 15 min at room temperature. The cells were washed by centrifugation, resuspended in 250 µl FACS buffer for analysis with BD FACSCanto II flow cytometer. Cell cycle analysis was performed with FlowJo.

### MT regrowth assay

MTs were depolymerized by incubating A549 cells grown on a 24-well plate on a custom-cut metal plate for 30 min on ice. MTs were repolymerized for 90 sec by incubating in 37°C medium. The cells were fixed immediately in cold methanol for 5 min. Cells were detected for MT asters by indirect immunofluorescence with anti-α-tubulin or anti-EB1 (microtubule plus-end binding protein 1) antibodies.

### Centrosome splitting analysis

A549 cells were fixed in cold methanol for 5 min and stained for γ-tubulin by indirect immunofluorescence and nuclei with DRAQ5. Confocal z-stack images were acquired with a 40× objective. The distance between γ-tubulin foci was measured on a maximal projection using ImageJ. Centrosomes were counted as split when the distance was more than 2 µm [52].

### Intensity measurements

Signal intensity of centrosomal ninein was measured from confocal images using ImageJ. Centrosomes were also identified by costaining with γ-tubulin.

### Plasmids

Constructs for EGFP-Rab7 and LAMP1-EGFP were provided by J. Gruenberg (University of Geneva, Geneva, Switzerland), NES-2×EGFP was provided by U. Greber (University of Zurich, Zurich, Switzerland) [62], HDAC8-Flag by Ed Seto (H. Lee Moffitt Cancer Center & Research Institute, Tampa, USA). LAMP1-mCherry plasmid was constructed by excising the EGFP cds of the LAMP1-EGFP plasmid with sites AgeI/BsrGI and replacing it with mCherry.

### Antibodies, chemicals and reagents

HB-65 was purchased from ATCC. The following antibodies were purchased: HDAC1, HDAC2, HDAC3 (obtained from BioVision), HDAC8, LAMP-1, C-Nap1, EB1 (Santa Cruz), giantin (Covance), EEA1 (Cell Signalling), γ-tubulin, Flag M2 (Sigma), acetylated α-tubulin, α-tubulin, ninein (Abcam). Stocks of nocodazole, taxol, dynasore, trychostatin A (obtained from Sigma), bafilomycin A (Calbiochem) were prepared in dmso (Calbiochem) and stored at −20°C. DRAQ5 was purchased from Biostatus Limited. Hoechst, R18, AF-labeled EGF, TF, WGA, and secondary antibodies were obtained from Invitrogen.

### Accession numbers for genes and proteins mentioned in text (NCBI Entrez Gene ID number)

*ATP6V1B2* (526); *C-Nap1* (11190); *EEA1* (8411); *Giantin* (2804); *HDAC1* (3065); *HDAC2* (3066); *HDAC3* (8841); *HDAC6* (10013); *HDAC8* (55869); *LAMP1* (3916); *Ninein* (51199); *Rab7* (7879); *Rootletin* (9696); *α-tubulin* (7846); *γ-tubulin* (7283).

## Supporting Information

Figure S1 **Efficiency of HDAC depletion and its effect on IAV X31 infection.** (A) Efficiency of HDAC depletion 72 h after transfection of siRNAs (HDAC1_6, HDAC1_1, HDAC1_3, HDAC3_1, HDAC3_2, HDAC3_4, HDAC8_2, HDAC8_4, obtained from Qiagen) in A549 cells. Protein levels were normalized to α-tubulin and quantified using ImageJ. (B) Specific depletion of a class I HDAC. Cellular mRNA levels of HDAC1, 3 and 8 following depletion with siRNAs HDAC1_6, HDAC3_4, HDAC8_2 were quantified. These 3 siRNAs were used for further experiments. Data are represented as mean ± SEM. (C)(D) Effect of depleting class I HDACs, vATPase subunit ATP6V1B2, on X31 infection in A549 (C) and HeLa ATCC (D) cells. Data are represented as mean ± SEM.
(TIF)

Figure S2 **MT orientation and Golgi localization following HDAC8 and rootletin depletion.** (A) Control (All*Neg), HDAC8-depleted (si HDAC8), rootletin-depleted (si rootletin) A549 cells, and control cells treated with 30 µM nocodazole for 30 min (All*Neg+nocodazole) were fixed for 5 min in cold methanol. Cells were stained for α-tubulin by IFA and nuclei with DRAQ5. Confocal z-stack images were acquired and maximally projected. Arrows indicate the MTOC. (B) Control (All*Neg), rootletin-depleted (si rootletin), HDAC8-depleted (si HDAC8) A549 cells were fixed for 5 min in cold methanol and stained by IFA with anti-LAMP1 antibody. Confocal z-stack images were acquired and maximally projected.
(TIF)

Figure S3 **Tubulin acetylation is decreased in HDAC8-depleted cells.** (A) A549 cells were depleted of HDAC1, 6, and 8 for 72 h. Cell lysates were subjected to Western blotting and detected for acetylated α-tubulin and α-tubulin. Acetylated α-tubulin protein levels were normalized to α-tubulin using ImageJ. (B) Control (All*Neg) and HDAC8-depleted (si HDAC8) A549 cells were fixed for 5 min in cold methanol and stained by IFA with anti-acetylated α-tubulin and anti-α-tubulin antibodies. Confocal z-stack images were acquired and maximally projected. The arrow indicates an MTOC.
(TIF)

Figure S4 **Localization of HDAC8.** A549 cells were transfected with a plasmid encoding HDAC8-Flag. The cells were fixed 20 h later and stained by indirect IFA with anti-HDAC8 (green) and anti-Flag M2 (red) antibodies. Confocal z-stacks were acquired and maximally projected.
(TIF)

Figure S5 **Co-depletion of HDAC1, 8 is efficient.** A549 cells were depleted of HDAC1, 8, and HDAC1/8, and subjected to Western blotting and detected for HDAC1, 8 and α-tubulin. HDAC1 and HDAC8 protein levels were normalized to α-tubulin using ImageJ.
(TIF)

Figure S6 **Trychostatin A specifically increases IAV X31 infection.** (A) A549 cells were treated with dmso or 5 µM TSA for 4 h, followed by X31 infection assay. Drugs were absent during infection. Data are represented as mean ± SEM. (B) A549 cells were treated with dmso or 5 µM TSA for 4 h, followed by infection assay with VSV, UUKV or X31. Data are represented as mean ± SEM.
(TIF)

Figure S7 **Rootletin is required for UUKV infection.** A549 cells were depleted of rootletin, ATP6V1B2, followed by UUKV infection assay. Data are represented as mean ± SEM.
(TIF)

Video S1 **HDAC8 promotes centripetal movement of endosomes containing internalized WGA.** Control (All*Neg) and HDAC8-depleted (si HDAC8) A549 cells grown in 96-well Matrix plates were incubated with imaging medium containing WGA-AF647 (5 µg/ml). After addition of WGA (t = zero), time-lapse images were acquired with the MD Assay Development 2 microscope at 15 min intervals up to 6 h, using a 20× objective. The video plays at 15 fps.
(MOV)
