# Supplementary figures and images for: Histone Deacetylase 8 Is Required for Centrosome Cohesion and Influenza A Virus Entry
Source: PLoS Pathog. 2011 Oct 27;7(10):e1002316. doi: 10.1371/journal.ppat.1002316 (PMC3203190; doi:10.1371/journal.ppat.1002316)

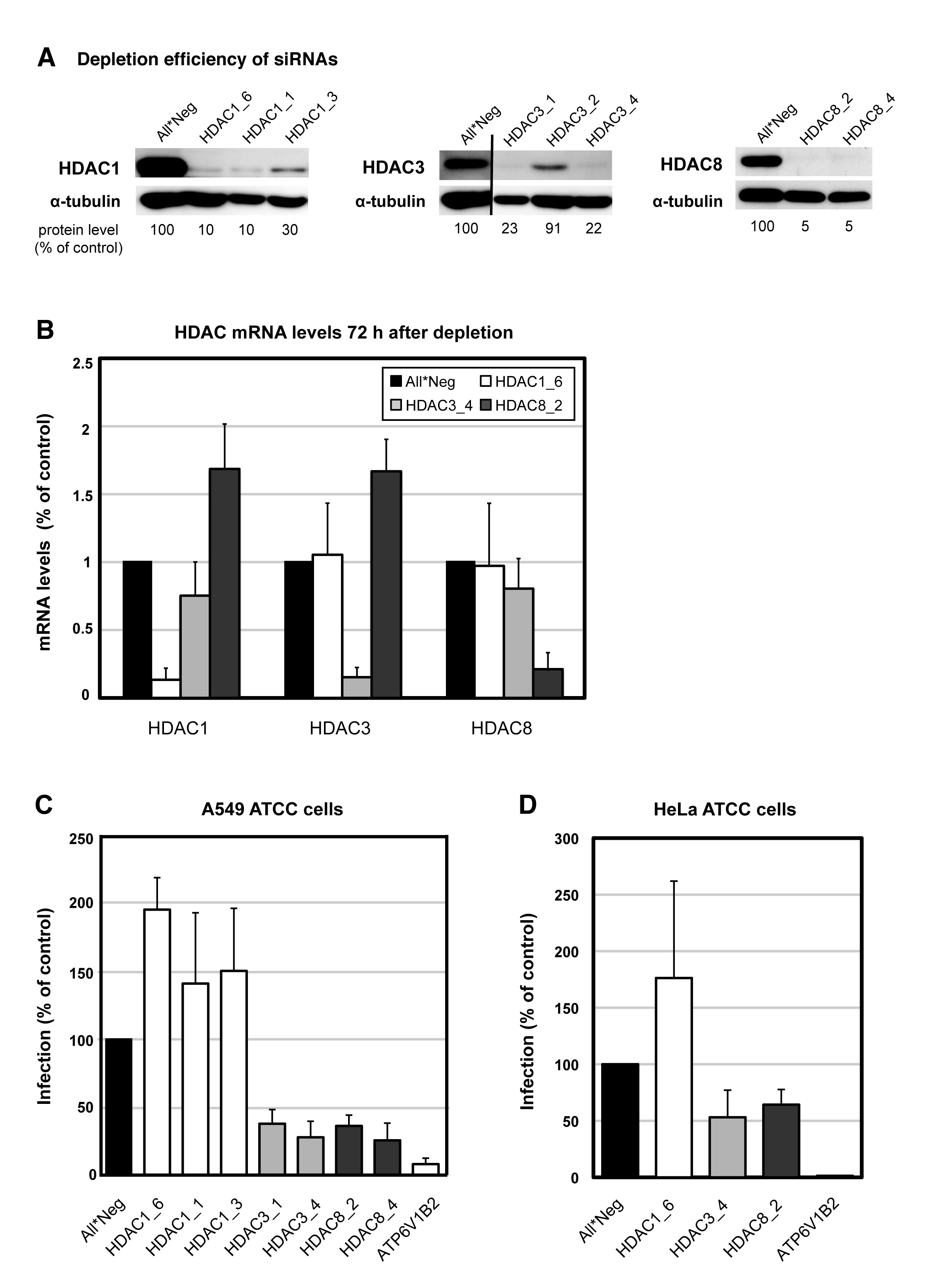

Supplement: Figure S1 — Efficiency of HDAC depletion and its effect on IAV X31 infection. (A) Efficiency of HDAC depletion 72 h after transfection of siRNAs (HDAC1_6, HDAC1_1, HDAC1_3, HDAC3_1, HDAC3_2, HDAC3_4, HDAC8_2, HDAC8_4, obtained from Qiagen) in A549 cells. Protein levels were normalized to α-tubulin and quantified using ImageJ. (B) Specific depletion of a class I HDAC. Cellular mRNA levels of HDAC1, 3 and 8 following depletion with siRNAs HDAC1_6, HDAC3_4, HDAC8_2 were quantified. These 3 siRNAs were used for further experiments. Data are represented as mean ± SEM. (C)(D) Effect of depleting class I HDACs, vATPase subunit ATP6V1B2, on X31 infection in A549 (C) and HeLa ATCC (D) cells. Data are represented as mean ± SEM. (TIF) [file ppat.1002316.s001.tif]

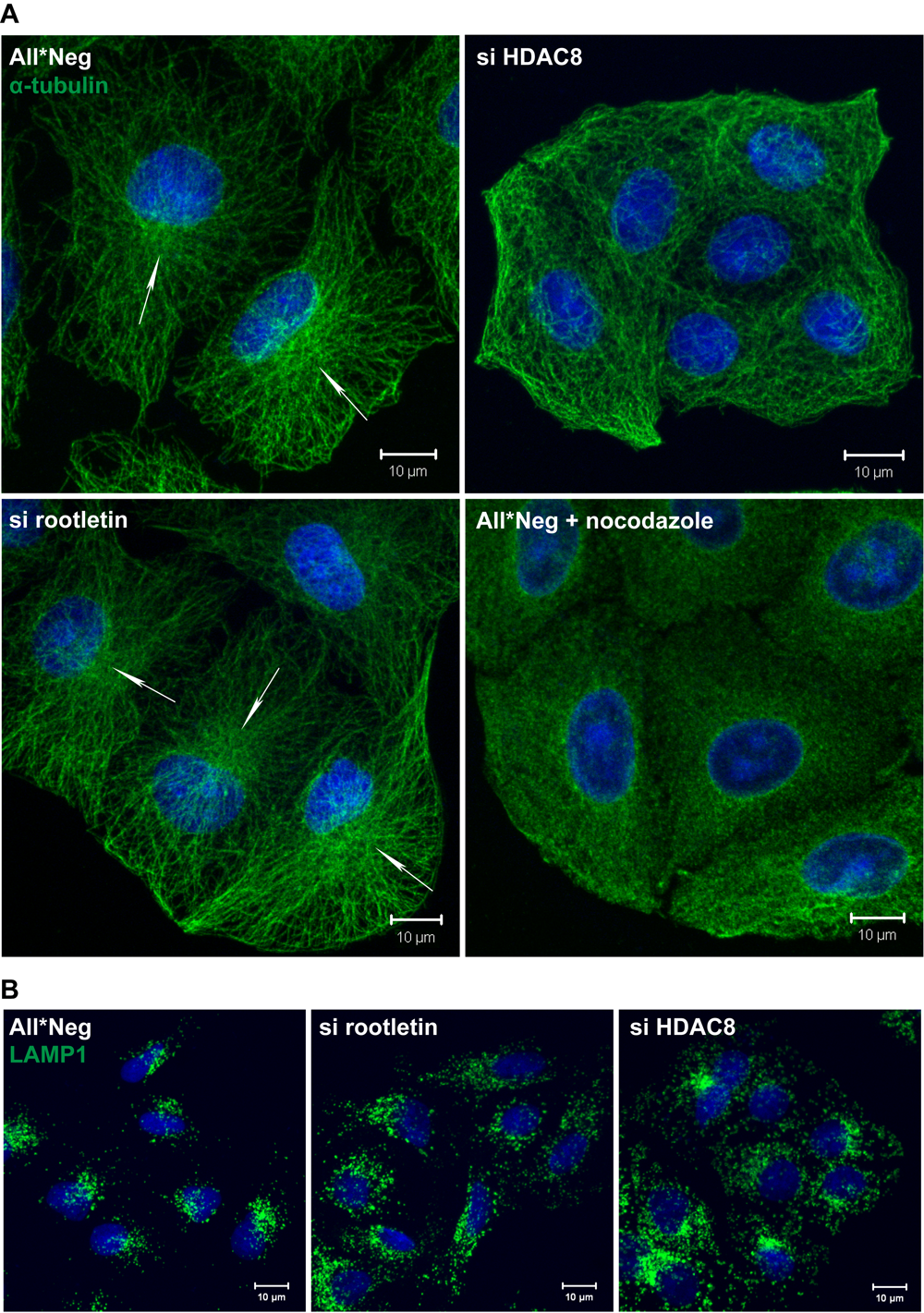

Supplement: Figure S2 — MT orientation and Golgi localization following HDAC8 and rootletin depletion. (A) Control (All*Neg), HDAC8-depleted (si HDAC8), rootletin-depleted (si rootletin) A549 cells, and control cells treated with 30 µM nocodazole for 30 min (All*Neg+nocodazole) were fixed for 5 min in cold methanol. Cells were stained for α-tubulin by IFA and nuclei with DRAQ5. Confocal z-stack images were acquired and maximally projected. Arrows indicate the MTOC. (B) Control (All*Neg), rootletin-depleted (si rootletin), HDAC8-depleted (si HDAC8) A549 cells were fixed for 5 min in cold methanol and stained by IFA with anti-LAMP1 antibody. Confocal z-stack images were acquired and maximally projected. (TIF) [file ppat.1002316.s002.tif]

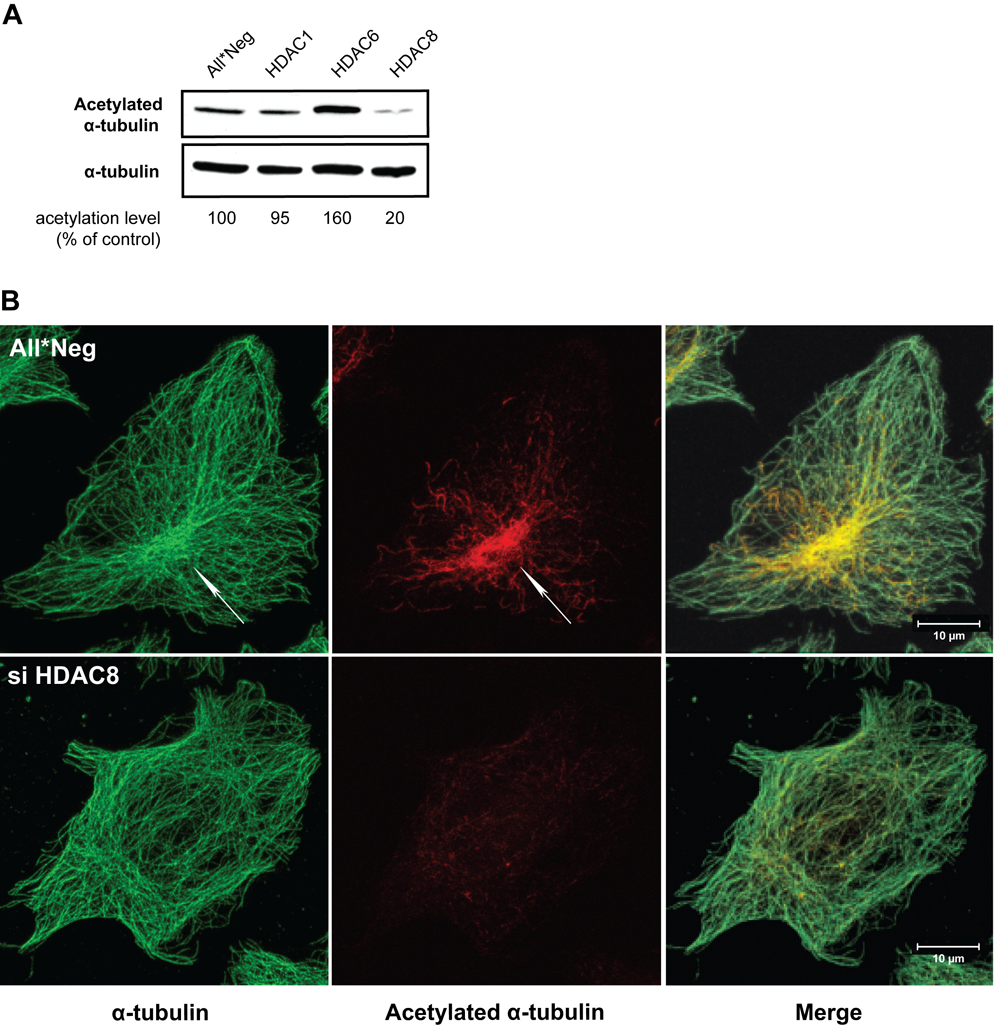

Supplement: Figure S3 — Tubulin acetylation is decreased in HDAC8-depleted cells. (A) A549 cells were depleted of HDAC1, 6, and 8 for 72 h. Cell lysates were subjected to Western blotting and detected for acetylated α-tubulin and α-tubulin. Acetylated α-tubulin protein levels were normalized to α-tubulin using ImageJ. (B) Control (All*Neg) and HDAC8-depleted (si HDAC8) A549 cells were fixed for 5 min in cold methanol and stained by IFA with anti-acetylated α-tubulin and anti-α-tubulin antibodies. Confocal z-stack images were acquired and maximally projected. The arrow indicates an MTOC. (TIF) [file ppat.1002316.s003.tif]

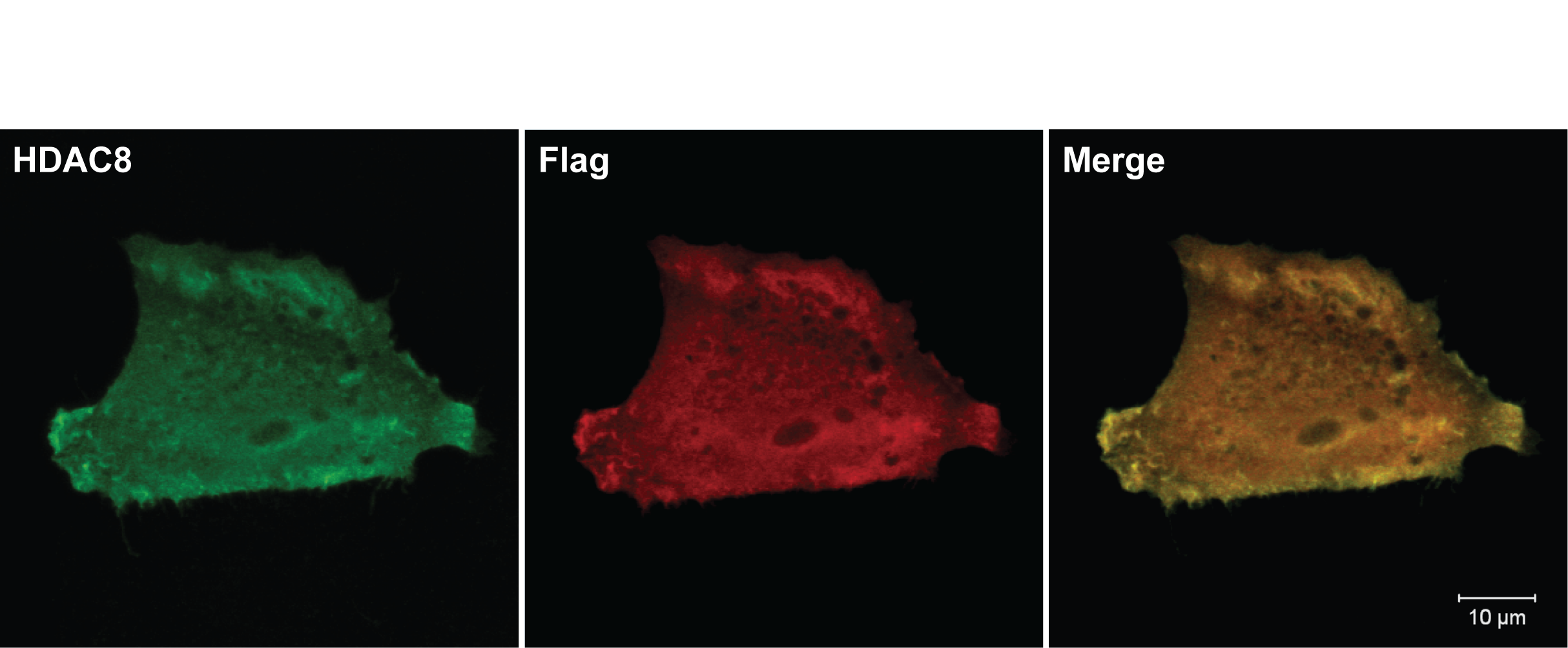

Supplement: Figure S4 — Localization of HDAC8. A549 cells were transfected with a plasmid encoding HDAC8-Flag. The cells were fixed 20 h later and stained by indirect IFA with anti-HDAC8 (green) and anti-Flag M2 (red) antibodies. Confocal z-stacks were acquired and maximally projected. (TIF) [file ppat.1002316.s004.tif]

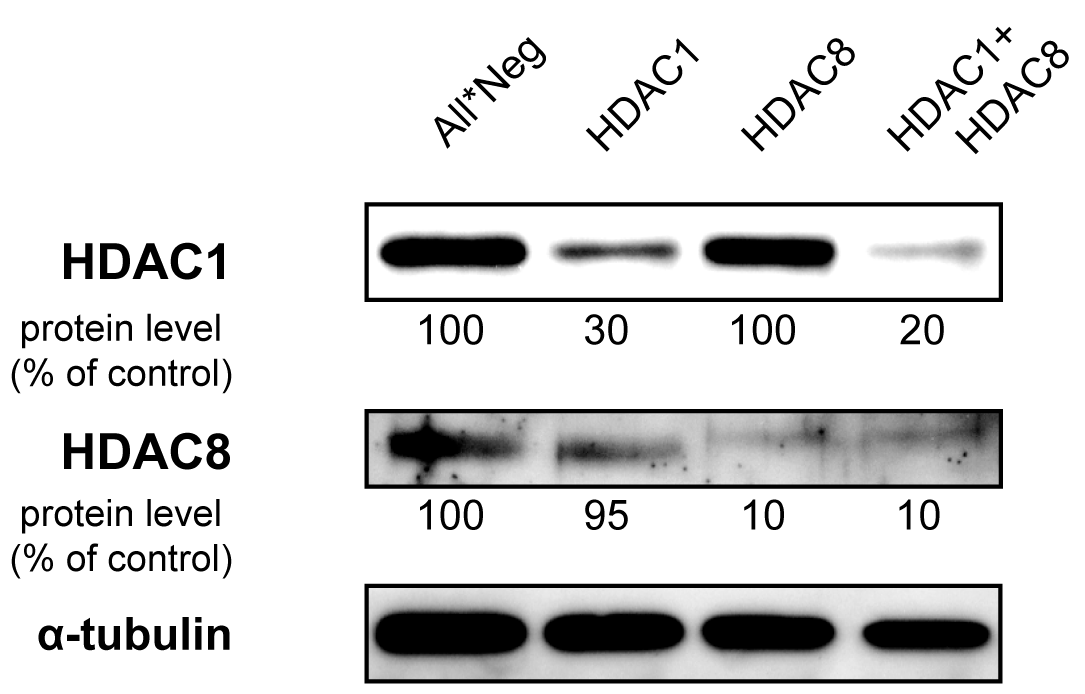

Supplement: Figure S5 — Co-depletion of HDAC1, 8 is efficient. A549 cells were depleted of HDAC1, 8, and HDAC1/8, and subjected to Western blotting and detected for HDAC1, 8 and α-tubulin. HDAC1 and HDAC8 protein levels were normalized to α-tubulin using ImageJ. (TIF) [file ppat.1002316.s005.tif]

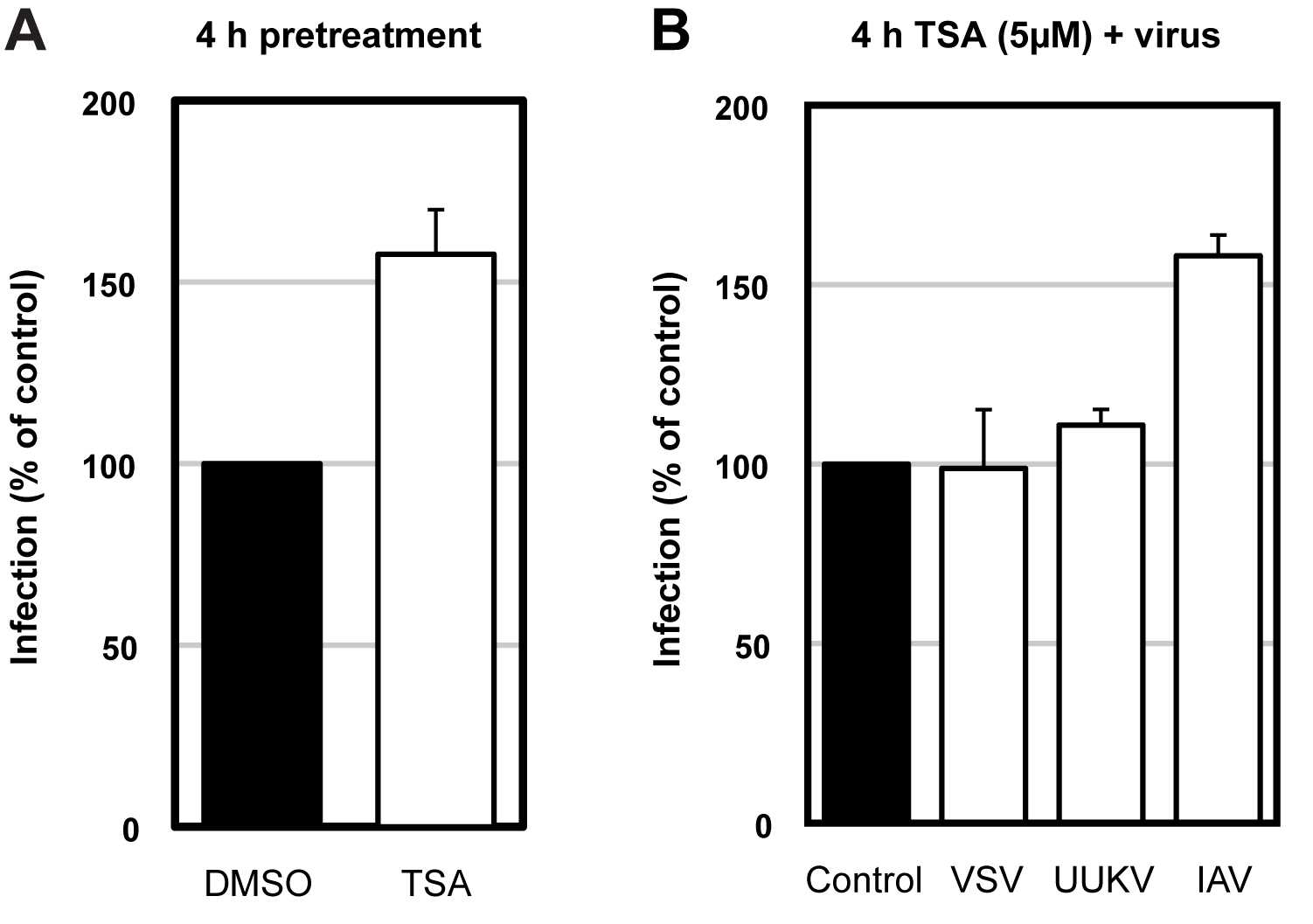

Supplement: Figure S6 — Trychostatin A specifically increases IAV X31 infection. (A) A549 cells were treated with dmso or 5 µM TSA for 4 h, followed by X31 infection assay. Drugs were absent during infection. Data are represented as mean ± SEM. (B) A549 cells were treated with dmso or 5 µM TSA for 4 h, followed by infection assay with VSV, UUKV or X31. Data are represented as mean ± SEM. (TIF) [file ppat.1002316.s006.tif]

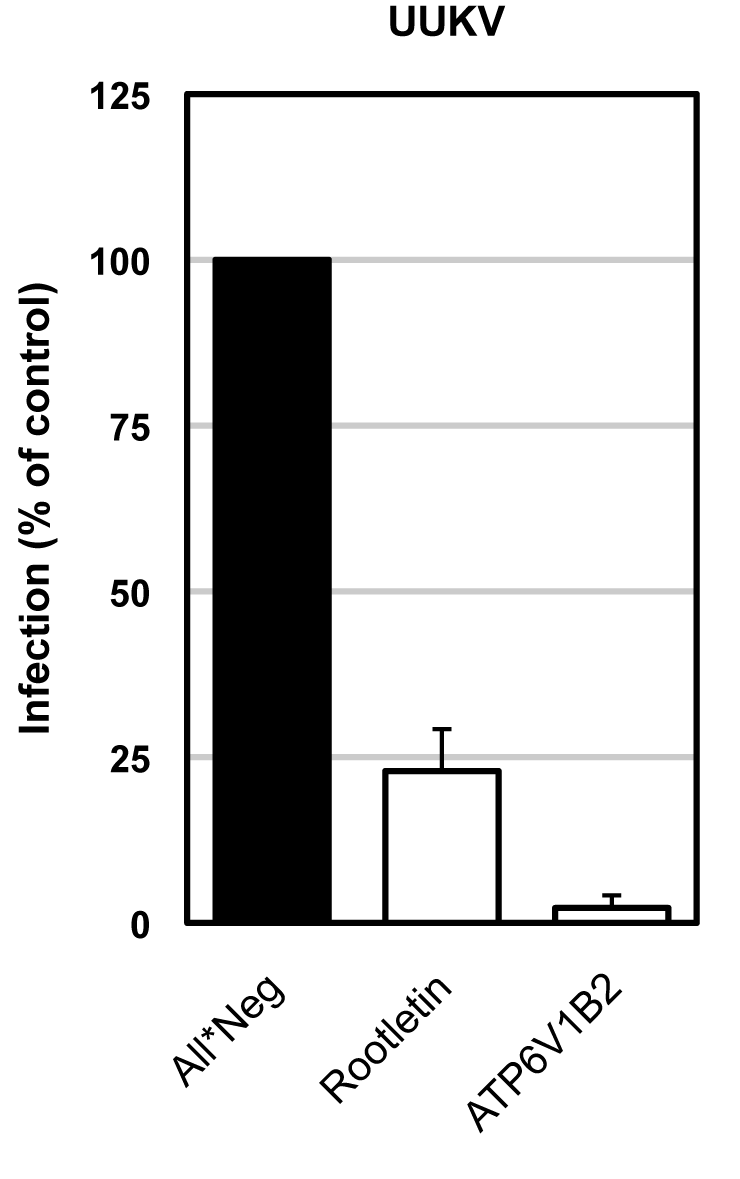

Supplement: Figure S7 — Rootletin is required for UUKV infection. A549 cells were depleted of rootletin, ATP6V1B2, followed by UUKV infection assay. Data are represented as mean ± SEM. (TIF) [file ppat.1002316.s007.tif]
